# MetaLIMS, a simple open-source laboratory information management system for small metagenomic labs

**DOI:** 10.1093/gigascience/gix025

**Published:** 2017-04-18

**Authors:** Cassie Elizabeth Heinle, Nicolas Paul Eugène Gaultier, Dana Miller, Rikky Wenang Purbojati, Federico M. Lauro

**Affiliations:** SCELSE, Nanyang Technological University, 60 Nanyang Dr, 637551, Singapore

**Keywords:** LIMS, customizable, GitHub, open-source, web application, sample management, JavaScript, php, mysql, HTML

## Abstract

**Background:** As the cost of sequencing continues to fall, smaller groups increasingly initiate and manage larger sequencing projects and take on the complexity of data storage for high volumes of samples. This has created a need for low-cost laboratory information management systems (LIMS) that contain flexible fields to accommodate the unique nature of individual labs. Many labs do not have a dedicated information technology position, so LIMS must also be easy to setup and maintain with minimal technical proficiency. **Findings:** MetaLIMS is a free and open-source web-based application available via GitHub. The focus of MetaLIMS is to store sample metadata prior to sequencing and analysis pipelines. Initially designed for environmental metagenomics labs, in addition to storing generic sample collection information and DNA/RNA processing information, the user can also add fields specific to the user's lab. MetaLIMS can also produce a basic sequencing submission form compatible with the proprietary Clarity LIMS system used by some sequencing facilities. To help ease the technical burden associated with web deployment, MetaLIMS options the use of commercial web hosting combined with MetaLIMS bash scripts for ease of setup. **Conclusions:** MetaLIMS overcomes key challenges common in LIMS by giving labs access to a low-cost and open-source tool that also has the flexibility to meet individual lab needs and an option for easy deployment. By making the web application open source and hosting it on GitHub, we hope to encourage the community to build upon MetaLIMS, making it more robust and tailored to the needs of more researchers.

## Introduction

MetaLIMS is a laboratory information management system (LIMS) for powerful but simple sample management. There are many varieties of LIMS at present, ranging from custom built options to out-of-the-box packages [1–9]. These options come in varying degrees of complexity and specificity, ranging from those that house sample names and metadata like MetaLIMS to those that track samples through a pipeline and analyze the data [1, 3–9]. The need for basic LIMS packages is increasing as sequencing costs drop, which enables smaller labs to initiate and manage larger sample collections [10, 11]. The result is a need for data management power that is greater than that of basic laboratory notebook and spreadsheets in order to manage samples. However, the combination of high prices, the time and complexity of setup and maintenance, and the need for flexibility to meet individual labs’ needs are often barriers to adopting LIMS systems, especially for smaller projects [1–9]. To help ease these three problems, MetaLIMS is an easy-to-set-up LIMS that is low in cost, open source, and has the ability to create custom fields for recording sample information. The goal of MetaLIMS is to allow more researchers to more easily and affordably manage their sample information.

## Findings

MetaLIMS differs from other open-source LIMS in that its focus is to store sample collection and processing metadata such as DNA extraction details prior to downstream sequencing and analysis pipelines. While many smaller labs outsource their sequencing or analysis to expertise outside their group, it is advantageous to have a LIMS that can record information detached from these sequencing and analysis pipelines. MetaLIMS was specifically designed for use by microbiology labs using high-throughput sequencing for metagenomic analysis. In addition to storing generic sample collection information and DNA/RNA processing information, the user can also add fields specific to the user's lab. MetaLIMS borrows the utility of web hosting services alongside MetaLIMS installation bash scripts to offer an installation process alongside its more advanced installation documentation to ease installation processes for users with milder computer experience.

## Database Description

**Figure 1:**
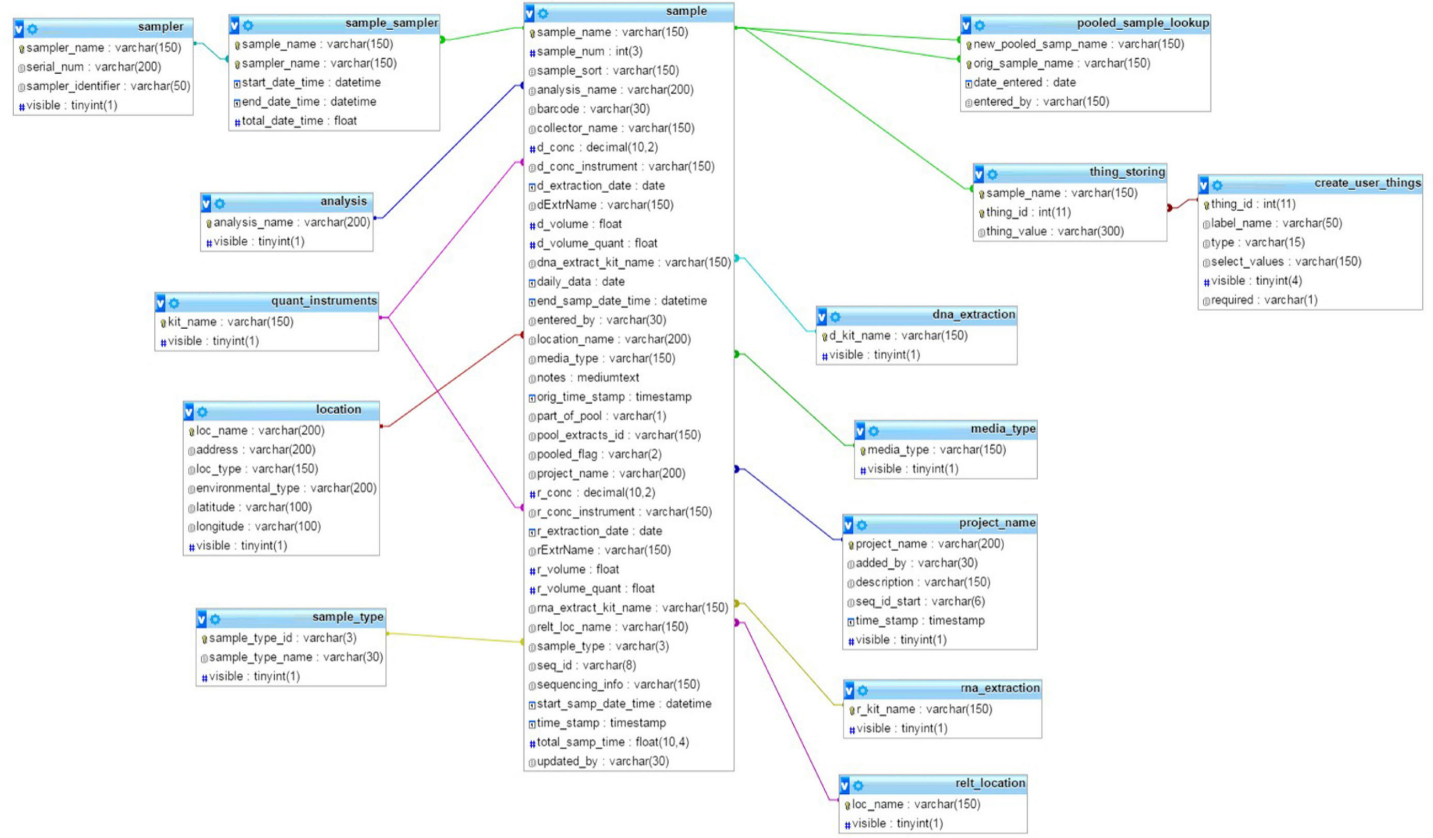
Database schema of MetaLIMS main sample tables. Please see Supplementary Fig. 1 for full database schema including auxiliary tables.

MetaLIMS functions using a web-based interface and was built and tested using XAMPP v. 3.2.1 and HTML5. MetaLIMS has been deployed on a production server using Apache 2.2.15, MySQL 5.5.43 (including mysqli module), and PHP 5.5.25 on a closed internet network. MetaLIMS can be deployed on any basic LAMPP stack using Apache 2.2.15, MySQL 5.5.43, PHP 5.5.25 (with mysqli extension), or newer. Figure 1 shows the database schema for MetaLIMS's main tables involved in sample recording. Full database schema can be found in Supplementary Fig. 1.

MetaLIMS uses many open-source packages such as Php Excel, DataTables, free html and php login templates, jquery libraries, and Creative Commons wallpapers that come packaged with the source code.

## Database Access and Deployment

MetaLIMS is free and easily downloaded or “cloned” from the project's public GitHub page (https://github.com/cheinle/MetaLIMS). A GitHub account is not required. Users can access the MetaLIMS installation and user manuals via the MetaLIMS GitHub wiki (https://github.com/cheinle/MetaLIMS/wiki). MetaLIMS allow users to customize the way that the database is hosted and backed up, and suggested options for new users can be found on the MetaLIMS wiki. Users will be able to implement their own database and web application security in a customized way, which is not possible with many commercial LIMS.

Due to the complexity of many LIMS, most laboratories need a dedicated information technology person(s) to set up and maintain their LIMS. In response to this problem, alongside MetaLIMS’ more advanced installation instructions, MetaLIMS suggests usage of hosted web services and MetaLIMS installation bash scripts to create simple and streamlined deployment and maintenance. Installation instructions were created utilizing one web hosting service, Amazon Lightsail. While MetaLIMS does not endorse these services, Lightsail was chosen due to Amazon's free one-month trial enabling users to freely try MetaLIMS on a hosted system [12]. MetaLIMS installation bash scripts can also be used for deployment of prerequisite LAMP stack and MetaLIMS application on any machine running Ubuntu 16.04 or Ubuntu 14.04. MetaLIMS bash scripts were tailored toward Ubuntu due to the large community of researchers using Ubuntu. Future work will involve extensibility of these scripts to other Linux and Unix distributions.

## User Interface

### Sample recording

**Figure 2:**
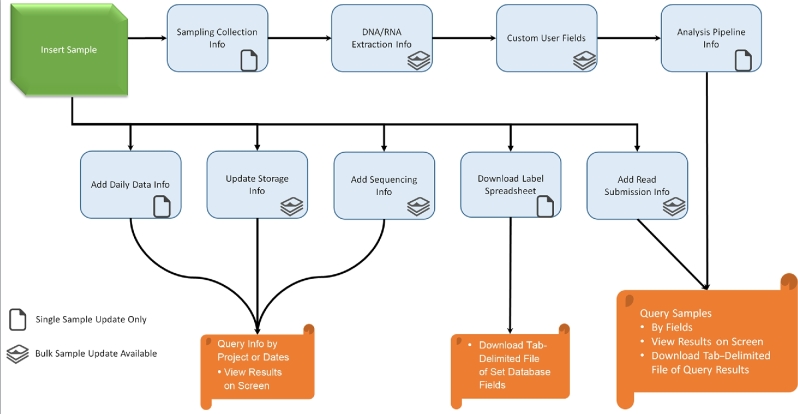
Sample recording workflow in MetaLIMS.

MetaLIMS was created to allow labs to easily manage their sample metadata by giving all lab members access to easily contribute, edit, and obtain sample information. Figure 2 shows the MetaLIMS workflow for recording and retrieving sample information. MetaLIMS contains fields to store basic sample information such as when samples were collected and what type of sampling collection method was used. It can also record DNA and RNA extraction information such as the kit used for extraction, which person performed the extraction, and the concentration and volume of extracted samples. MetaLIMS can store sample storage information for other downstream events such as sequencing submission information and which analysis pipeline was used.

Users can access sample information one record at a time using the “Update Sample” function. Here the user can get a detailed view of a particular entry and make edits or additions as necessary.

**Figure 3:**
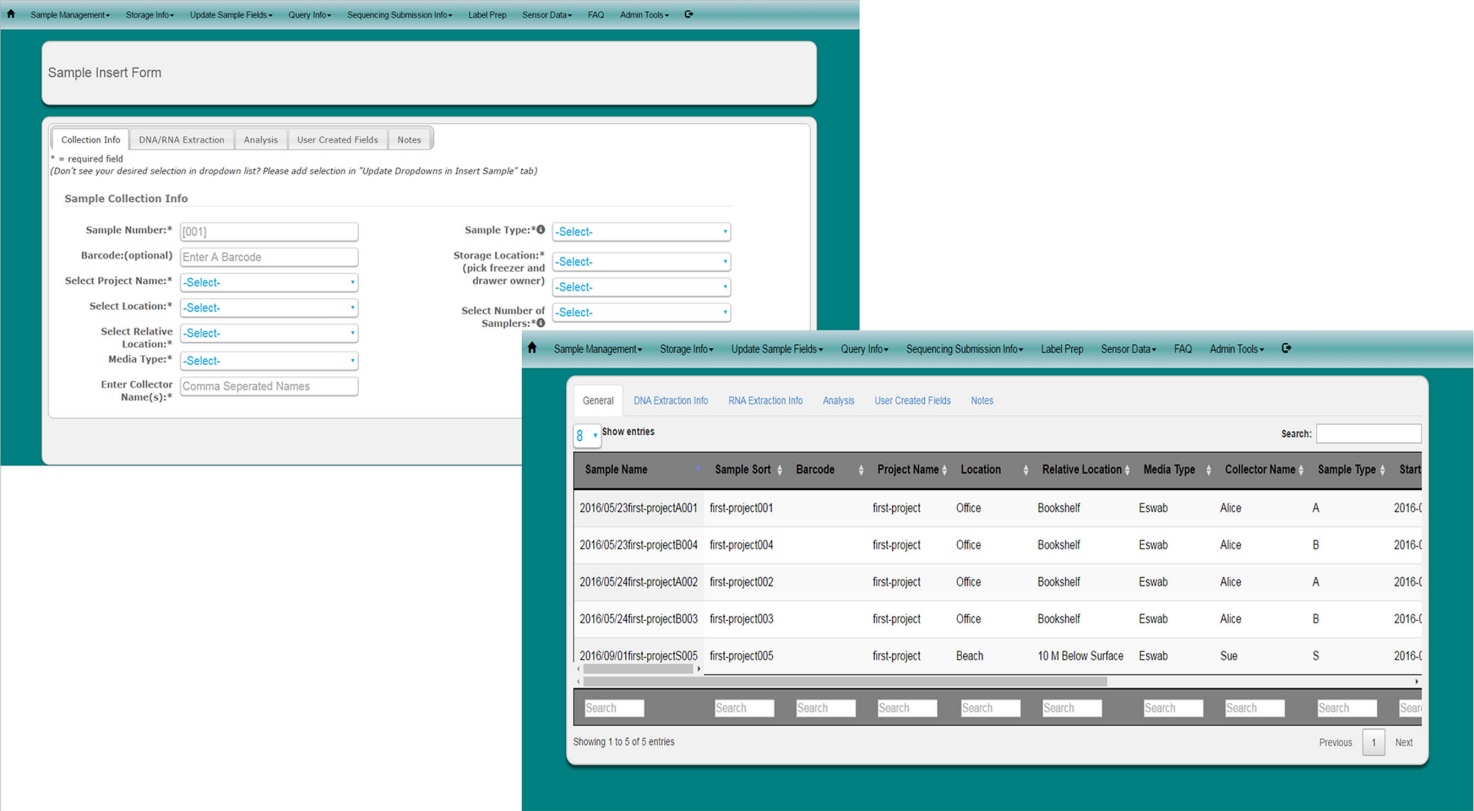
MetaLIMS sample input.

Users can also view information for batches of samples by using the “Query Info” function to view samples by date or by specific sample collection or processing criteria. Users will be able to view their sample records on the screen or download selected records as a tab-delimited document for further exploration and data manipulation. Figure 3 shows the sample input form and output for sample queries for MetaLIMS.

### Custom recording

Custom fields may be created by the admin user for users to enter additional sample information unique to an individual lab or project. Admin users are able to add custom text entries (as either free text or dropdown boxes) as well as numeric entries. Admin users will be able to indicate which custom entries should be required entries for each sample. These new custom fields appear in the tab “User Created Fields” on both the sample entry and sample update forms.

MetaLIMS can also record daily averages of any user-specified data under the “Sensor Data” feature. This allows for custom recording of any sensor data by day and location. For example, this would allow a user to store measurements as metadata for a set of samples, such as temperature, humidity, or light intensity.

Using these two functions would allow the lab to track and view any desired information that can be measured for either individual samples or a batch of samples.

### Sequencing sample sheet

MetaLIMS was originally built to generate output of a sequencing submission form that is ready for downstream entry into a Genologics Clarity LIMS Gold pipeline but has been adapted to output the sample submission form for the generic Clarity LIMS sample sheet used in the Clarity LIMS Silver and Run Manager versions [1]. Because of the variety of types of sequencing submission forms used by different sequencing facilities, additional customization of MetaLIMS by users with more advanced technical skills may be required to generate sequencing submission spreadsheets in the format required by a specific sequencing facility.

### Data submission

For posterity, researchers who upload sequencing data to public databases can use the read submission function to keep a record of if sequencing files associated with a specific sample or set of samples have been submitted to repositories such as DDBJ, Genbank, or ENA. This does not create the submission but stores the information as record keeping for the user. Users can enter the read submission name, date submitted, and type of experiment the sample was included in.

### Labels

MetaLIMS gives the user the ability to print out labels, ensuring consistent sample naming and labeling within a lab. This helps prevent problems such as illegible or smeared handwriting, confusing date formats, and vague or redundant sample naming. MetaLIMS allows the user to print out labels containing sample names, sampling date and time, project name, sample type, and sample number, thus making the tube labeling unambiguous.

MetaLIMS can work with common desktop label printers for researchers looking to print labels. This label function can generate a form of QR codes for either sample names or other sample information for barcoding if a “barcode” field is populated for these samples. Alternatively, a tab-delimited file can be downloaded for easy uploading to common label-making software. These common label makers allow users to connect to a “database” such as an Excel worksheet, comma-separated text file, or tab-delimited file for text and barcode generation [13, 14].

## Database Application

MetaLIMS is intended as a sample management solution for smaller labs as the responsibility of creating and storing larger amounts of data comes to smaller research groups. MetaLIMS is currently in use by the Air Microbiome Group with the Singapore Centre for Environmental Life Sciences Engineering to house sample information from sample collection as well as archiving information on downstream processes performed such as data analysis, sequencing, and read submission to public databases [15]. While there is increasing growth in the number of LIMS being created to try and fill the unique needs of various labs, Table 1 shows a comparison of MetaLIMS to four popular open-source LIMS, MISO LIMS [5], BIKA-LIMS [6], SIERRA LIMS [7], and MendeLIMS [8], to help define MetaLIMS for user usage.

**Table 1.** Comparison of MetaLIMS to popular open-source LIMS

LIMS Software	Miso LIMS	Bika LIMS	Sierra LIMS	MendeLIMS	MetaLIMS
For NGS sequencing	Yes	Not specific	Yes	Yes	No
For medical sample	Not specific	Not specific	Not specific	Yes	No
For sample metadata	Pre-defined fields and free-form “notes”; sample type configuration for advanced users	Allows sample types	Only sequencing metadata	Yes, for clinical samples	Yes
Input sample types	Extracted DNA	Not specific	Extracted DNA/RNA	Clinical samples	Environmental samples
Customizable—user can create fields	Can add sequencers	Yes	No	Can populate some configurable fields and dropdowns	Yes
Web based	Yes	No	Yes	Yes	Yes
Software	JDK7, Tomcat 8, MySQL 5, Flyway, Maven, git	Python	Perl	Javascript, Ruby	PHP
Database	MySQL	ZODB (expected PostgreSQL intergration)	MySQL	MySQL, PostgreSQL, or SQLLite	MySQL
Computer skills [16]	Advanced	Medium	Basic	Basic	Basic
Website	http://tgac.github.io/miso-lims/	https://github.com/bikalabs/bika.lims	http://www.bioinformatics.babraham.ac.uk/projects/sierra/	http://dna-discovery.stanford.edu/software/mendelims/	https://github.com/cheinle/MetaLIMS/wiki

MetaLIMS differs in comparison to other LIMS in that unlike many LIMS, which were created to store and track samples through next-generation sequencing (NGS) pipelines, such as MISO, SIERRA, BIKA-LIMS, or specific to medical use such as MendeLIMS or BIKA-HEALTH, MetaLIMS is defined specifically for the use of storing sample metadata prior to high-throughput sequencing pipelines and analysis. While some LIMS offer the flexibility of adding custom fields or custom population dropdown fields, MetaLIMS offers this capability without the extra bulk of storing downstream sequencing library prep and machine metrics, which may not be needed by small labs that do not do their own sequencing. Lastly, while all LIMS compared grant the fluid adaptability of open-source software, many still require extensive Unix or command-line interface knowledge to deploy.

## Conclusions

The decrease in cost of sequencing has led to a subsequent increase in the initiation and management of large data collection by small labs. This increase in the influx in data generated by such projects creates a need for more powerful sample management tools than traditional lab notebooks and spreadsheets. MetaLIMS is able to offer labs easy sharing and access to recorded sample information across lab members. MetaLIMS is a unique solution that is free and customizable for small metagenomic labs that wish to store metagenomic sample collection and processing information but do not need the extra bulk of recording NGS or analysis data, which is common in many NGS LIMS. MetaLIMS has demonstrated that it overcomes key challenges often associated with LIMS by being free of cost and open source and having customizable sample-specific fields to add flexibility to meet the unique needs of different labs. By building MetaLIMS on a common web platform and offering a solution for easy deployment through web hosting, the complexity of deploying and managing a web application becomes minimal and MetaLIMS becomes easy to set up and maintain. It is our further desire that making the web application open source and hosting it on GitHub will encourage the community to both utilize and build upon MetaLIMS, allowing it to become more robust and tailored toward the community's growing needs.

## Abbreviation

LIMS: Laboratory Information Management System.

## Supplementary Material

GIGA-D-17-00035_Original_Submission.pdf

GIGA-D-17-00035_Revision_1.pdf

GIGA-D-17-00035_Revision_2.pdf

Response_to_reviewer_comments_Original_Submission.pdf

Response_to_reviewer_comments_Revision_1.pdf

Reviewer_1_Report_(Original_Submission).pdf

Supplemental material

## Availability of supporting data

Snapshots of the supporting code are available from the GigaScience GigaDB repository [17].

## Availability of supporting source code and requirements

Use of MetaLIMS, its data, and source code (https://github.com/cheinle/MetaLIMS) are unrestricted for use by academic and commercial researchers.

- Project name: MetaLIMS, A Simple Open-Source Laboratory Information Management System for Small Metagenomic Labs
- Project home page: https://github.com/cheinle/MetaLIMS
- Operating system(s): deployment—Linux, Access—platform independent
- Programming language: PHP 5.5.25 (including mysqli module)
- Other requirements: Chrome (version 47.0.2526.111) or Firefox (43.0.4; preferred), Apache 2.2.15, 266 HTML5, MySQL 5.5.43 (no STRICT_TRANS_TABLES mode)
- License: MetaLIMS is released under the GNU General Public License
- Any restrictions to use by non-academics: none
