# Supplementary material for: MetaLIMS, a simple open-source laboratory information management system for small metagenomic labs
Source: Gigascience. 2017 Apr 18;6(6):1–6. doi: 10.1093/gigascience/gix025 (PMC5449644; doi:10.1093/gigascience/gix025)
Supplement: GIGA-D-17-00035_Original_Submission.pdf [file gix025_GIGA-D-17-00035_Original_Submission.pdf]

Manuscript

Click here to download Manuscript metalims_manuscript_cheinle.pdf

Title page

MetaLIMS, A Simple Open-Source Laboratory Information Management System for Small Metagenomic Labs

Cassie Elizabeth Heinle (corresponding author)
cheinle@ntu.edu.sg
SCELSE
Nanyang Technological University
60 Nanyang Dr, 637551

Nicolas Paul Eugene Gaultier
GNPEugene@ntu.edu.sg
SCELSE
Nanyang Technological University
60 Nanyang Dr, 637551

Dana Miller
dana.miller@ntu.edu.sg
SCELSE
Nanyang Technological University
60 Nanyang Dr, 637551

Ricky Wenang Purbojati
rpurbojati@ntu.edu.sg
SCELSE

Nanyang Technological University
60 Nanyang Dr, 637551

Federico M. Lauro
FLauro@ntu.edu.sg
SCELSE-NTU
Nanyang Technological University
60 Nanyang Dr, 637551

Abstract

Background

As the cost of sequencing continues to fall, smaller groups increasingly initiate and manage larger sequencing projects and take on the complexity of data storage for high volumes of samples. This has created a need for low-cost laboratory information management systems (LIMS) that contain flexible fields to accommodate the unique nature of individual labs. Many labs do not have a dedicated IT position, so LIMS must also be easy to setup and maintain with minimal technical proficiency.

Findings

MetaLIMS is a free and open source web-based application available via GitHub. The focus of MetaLIMS is to store sample metadata prior to sequencing and analysis pipelines. Initially designed for environmental metagenomics labs, in addition to storing generic sample collection information and DNA/RNA processing information the user can also add fields specific to the users lab. MetaLIMS can also produce a basic sequencing submission form compatible with the proprietary Clarity LIMS system used by some sequencing facilities. To help ease the technical burden associated with web-deployment, MetaLIMS options the use of commercial web-hosting combined with MetaLIMS bash scripts for ease of set-up.

Conclusions

MetaLIMS overcomes key challenges common in LIMS by giving labs access to a low-cost and open source tool that also has the flexibility to meet individual lab needs and an option for easy deployment. By making the web application open source and hosting it on GitHub, we hope to encourage the community to build upon MetaLIMS, making it more robust and tailored to the needs of more researchers.

Keywords

LIMS-customizable-GitHub-open source-web application-sample management-javascript-php-mysql-html

Background

MetaLIMS is a Laboratory Information Management System (LIMS) for powerful but simple sample management. There are many varieties of LIMS at present, ranging from custom built options to out-of-the-box packages [1-9]. These options come in varying degrees of complexity and specificity starting from those which house sample names and metadata like MetaLIMS, to those that track samples through a pipeline and analyze the data [1][3-9]. The need for basic LIMS packages is increasing as sequencing costs drop, which enables smaller labs to initiate and manage larger sample collections [10][11]. The result is a need for data management power that is greater than that of basic laboratory lab notebook and spreadsheets in order to manage samples. However, the combination of high prices, the time and complexity of set-up and maintenance, and the need for flexibility to meet individual labs' needs are often barriers to adopting LIMS systems, especially for smaller projects [1-9]. To help ease these three problems, MetaLIMS is an easy-to-set-up LIMS that is low in cost, open-source, and has the ability to create custom fields for recording sample information. The goal of MetaLIMS is to allow more researchers to more easily and affordably manage their sample information.

Findings

MetaLIMS differs from other open-source LIMS in that its focus is to store sample collection and processing metadata such as DNA extraction details, prior to downstream sequencing and analysis pipelines. While many smaller labs outsource their sequencing or analysis to expertise outside their group, it is advantageous to have a LIMS which can record information detached from these sequencing and analysis pipelines. MetaLIMS was specifically designed for use by microbiology labs using high throughput sequencing for metagenomic analysis. In addition to storing generic sample collection information and DNA/RNA processing information, the user can also add field's specific to the users' lab. MetaLIMS borrows the utility of web hosting services alongside MetaLIMS installation bash scripts to offer an installation process alongside its more advanced installation documentation to ease installation

processes for users with milder computer experience.

Database Description

MetaLIMS functions using a web based interface and was built and tested using XAMPP v3.2.1 and HTML5. MetaLIMS has been deployed on a production server using Apache 2.2.15 , MySQL 5.5.43 (including mysqli module), PHP 5.5.25 on a closed internet network. MetaLIMS can be deployed on any basic LAMPP stack using Apache 2.2.15 , MySQL 5.5.43, PHP 5.5.25 (with mysqli extension) or newer. Figure 1 shows the database schema for MetaLIMS's main tables involved in sample recording. Full database schema can be found in Supplementary Figure 1.

Figure 1. Database schema of MetaLIMS main sample tables. Please see supplementary Figure 1 for full database schema including auxiliary tables

MetaLIMS uses many open source packages such as Php Excel, DataTables, free html and php login templates, jquery libraries, and Creative Commons wallpapers which come packaged with the source code.

Database access and Deployment

MetaLIMS is free and easily downloaded or 'cloned' from the project's public GitHub page (https://github.com/cheinle/MetaLIMS). A GitHub account is not required. Users can access the MetaLIMS installation and user manuals via the MetaLIMS GitHub wiki (https://github.com/cheinle/MetaLIMS/wiki) . MetaLIMS allow users to customize the way that the database is hosted and backed-up and suggested options for new users can be found on the MetaLIMS wiki. Users will be able to implement their own database and web application security in a custom way which is not possible with many commercial LIMS.

Due to the complexity of many LIMS, most laboratories need a dedicated IT person(s) to set-up and maintain their LIMS. In response to this problem, alongside MetaLIMS more advanced installation instructions, MetaLIMS suggests usage of hosted web services and MetaLIMS installation bash scripts to create simple and streamline deployment and maintenance. Installation instructions were created utilizing one web hosting service, Amazon Lightsail. While MetaLIMS does not endorse these services, Lightsail was chosen due to Amazon's free one month trial enabling users to freely try MetaLIMS on a hosted system [12]. MetaLIMS installation bash scripts can also be used for deployment of prerequisite LAMP stack and MetaLIMS application on any machine running Ubuntu 16.04 or Ubuntu 14.04. MetaLIMS bash scripts were tailored towards Ubuntu due to the large community of researchers using Ubuntu. Future work will involve extensibility of these scripts to other Linux and Unix distributions.

User Interface

Sample recording

MetaLIMS was created to allow labs to easily manage their sample metadata by giving all lab members access to easily contribute, edit, and obtain sample information. Figure 2 shows the MetaLIMS workflow for recording and retrieving sample information. MetaLIMS contains fields to store basic sample information such as when samples were collected and what type of sampling collection method was used. It can also record DNA and RNA extraction information such as the kit used for extraction, which person performed the extraction, and the concentration and volume of extracted samples. MetaLIMS can store sample storage information for other downstream events such as sequencing submission information and which analysis pipeline was used.

Figure 2. Sample recording workflow in MetaLIMS

Users can access sample information one record at a time using the 'Update Sample' function. Here the user can get a detailed view of a particular entry and make any edits or additions as necessary.

Users can also view information for batches of samples by using the ‘Query Info’ function to view samples by date or by specific sample collection or processing criteria. Users will be able to view their sample records on the screen or download selected records as a tab-delimited document for further exploration and data manipulation. Figure 3 shows the sample input form and output for sample queries for MetaLIMS.

Figure 3. MetaLIMS sample input

Custom recording

Custom fields may be created by the admin user for users to enter additional sample information unique to an individual lab or project. Admin users are able to add custom text entries (as either free text or drop down boxes) as well as numeric entries. Admin users will be able to indicate which custom entries should be required entries for each sample. These new custom fields appear in the tab ‘User Created Fields’ on both the sample entry and sample update forms.

MetaLIMS can also record daily averages of any user-specified data under the ‘Sensor Data’ feature. This allows for custom recording of any sensor data by day and location. For example this would allow a user to store measurements as metadata for a set of samples, such as temperature, humidity, or light intensity.

Using these two functions would allow the lab to track and view any desired information that can be measured for either individual samples or a batch of samples.

Sequencing sample sheet

MetaLIMS was originally built to generate output of a sequencing submission form that is ready for downstream entry into a Genologics Clarity LIMS Gold pipeline but has been adapted to output the sample submission form for generic Clarity LIMS sample sheet used in the Clarity LIMS Silver and Run Manager versions [1]. Because of the variety of types of sequencing submission forms used by different

sequencing facilities, additional customization of MetaLIMS by users with more advanced technical skills may be required to generate sequencing submission spreadsheets in the format required by a specific sequencing facility.

Data submission

For posterity, researchers who upload sequencing data to public databases can use the read submission function to keep a record of if sequencing files associated with a specific sample or set of samples have been submitted to repositories such as DDBJ, Genbank,or ENA. This does not create the submission, but stores the information as record keeping for the user. Users can enter the read submission name, date submitted, and type of experiment the sample was included in.

Labels

MetaLIMS gives the user ability to print out labels, ensuring consistent sample naming and labeling within a lab. This helps prevent problems such as illegible or smeared hand-writing, confusing date formats, and vague or redundant sample naming. MetaLIMS allows the user to print out labels containing sample names, sampling date and time, project name, sample type, and sample number, thus making the tube labeling unambiguous.

MetaLIMS can work with common desktop label printers for researchers looking to print labels. This label function can generate a form of QR codes for either sample names or other sample information for barcoding if a ‘barcode’ field is populated for these samples. Alternatively, a tab-delimited file can be downloaded for easy uploading into to common label making software. These common label makers allow users to connect to a ‘database’ such as an excel worksheet, comma-separated text file, or tab-delimited file for text and barcode generation [13][14].

Database application

MetaLIMS is intended as a sample management solution for smaller labs as the responsibility of creating and storing larger amounts of data comes to smaller research groups. MetaLIMS is currently in use by the Air Microbiome group with the Singapore Centre for Environmental Life Sciences Engineering to house sample information from sample collection as well as archiving information on downstream processes performed such as data analysis, sequencing, and read submission to public databases [15]. While there is increasing growth in the number of LIMS being created to try and fill the unique needs of various labs, Table 1 shows a comparison of MetaLIMS to 4 popular open-source LIMS, MISO LIMS [5], BIKA-LIMS [6] ,SIERRA LIMS [7], and MendeLIMS [8] to help define MetaLIMS for user usage.

MetaLIMS differs in comparison to other LIMS in that unlike many LIMS which were created to store and track samples through NGS sequencing pipelines, such as MISO, SIERRA,BIKA-LIMS, or specific for medical use such as Mendel or BIKA-HEALTH, MetaLIMS is defined specifically for use of storing sample meta-data prior to high throughput sequencing pipelines and analysis. While some LIMS offer the flexibility of adding custom fields or custom population of dropdown fields, MetaLIMS offers this capability without the extra bulk of storing downstream sequencing library prep and machine metrics which may not be needed by small labs which do not do their own sequencing. Lastly, while all LIMS compared grant the fluid adaptability of open-source software, many still require extensive unix or command-line interface knowledge to deploy.

LIMS Software	Miso LIMS	Bika LIMS	Sierra LIMS	MendeLIMS	MetaLIMS
**For NGS sequencing**	Yes	Not specific	Yes	Yes	No
**For medical sample**	Not specific	Not specific	Not specific	Yes	No
**For sample metadata**	Only add at sample creation	Allows sample types	Only sequencing metadata	Yes, for clinical samples	Yes
**Input sample types**	Extracted DNA	Not specific	Extracted DNA/RNA	Clinical samples	Environmental samples
**Customizable - User can create fields**	Can add sequencers	Yes	No	Can populate some configurable fields and dropdowns	Yes
**Web Based**	Yes	No	Yes	Yes	Yes
**Software**	JDK7, Tomcat 8, MySQL 5, Flyway, Maven, git, Eclipse	Python	Perl	Javascript, Ruby	PHP
**Database**	MySQL	ZODB (expected PostgreSQL intergration)	MySQL	MySQL, PostgreSQL, or SQLLite	MySQL
**Computer skills [16]**	Advanced	Medium	Basic	Basic	Basic
**Website**	http://www.earlham.ac.uk/miso	https://github.com/bikalabs/bika.lims	http://www.bioinformatics.babraham.ac.uk/projects/sierra/	http://dna-discovery.stanford.edu/software/mendelims/	https://github.com/cheinle/MetaLIMS/wiki

Table 1. Comparison of MetaLIMS to popular open-source LIMS

Conclusions

The decrease in cost of sequencing has led to a subsequent increase in the initiation and management of large data collection by small labs. This increase in the influx in data generated by such projects creates a need for more powerful sample management tools than traditional lab notebooks and spreadsheets. MetaLIMS is able to offer labs easy sharing and access of recorded sample information across lab members. MetaLIMS is a unique solution which is free and customizable for small metagenomic labs which wish to store metagenomic sample collection and processing information but do

not need the extra bulk of recording NGS sequencing or analysis data, which is common in many NGS LIMS. MetaLIMS has demonstrated it overcomes key challenges often associated with LIMS by being free of cost and open source and having customizable sample specific fields to add flexibility to meet the unique needs of different labs. By building MetaLIMS on a common web platform and offering a solution for easy deployment through web-hosting, the complexity of deploying and managing a web application becomes minimal and MetaLIMS becomes easy to set-up and maintain. It is our further desire that making the web application open source and hosting it on GitHub that it will encourage the community to both utilize and build upon MetaLIMS allowing it to become more robust and tailored towards the community's growing needs.

Availability of supporting source code and requirements

Use of MetaLIMS, its data, and source code https://github.com/cheinle/MetaLIMS are unrestricted for use by academic and commercial researchers.

- Project name: MetaLIMS, A Simple Open-Source Laboratory Information Management System for Small Metagenomic Labs
- Project home page: https://github.com/cheinle/MetaLIMS
- Operating system(s): Deployment – Linux, Access- Platform independent
- Programming language: PHP 5.5.25 (including mysqli module)
- Other requirements: Chrome (Version 47.0.2526.111) or Firefox (43.0.4) (preferred), Apache 2.2.15, 266 HTML5, MySQL 5.5.43 (no STRICT_TRANS_TABLES mode)
- License: MetaLIMS is released under the GNU General Public License
- Any restrictions to use by non-academics: None

Declarations

List of abbreviations

AMI: Amazon Machine Image

AWS: Amazon Web Services

LIMS: Laboratory Information Management System

VPS: Virtual Private Server

Ethics approval and consent to participate

Note applicable

Consent for publication

Note applicable

Competing interests

The authors declare that they have no competing interests.

Funding

Singapore Ministry of Education Academic Research Fund Tier 3 MOE2013-T3-1-013, and the Singapore Centre for Environmental Life Sciences Engineering (SCELSE), whose research is supported by the National Research Foundation Singapore, Ministry of Education,Nanyang Technological University, and National University of Singapore, under its Research Centre of Excellence Program.

Authors' contributions

CH implemented the package and wrote the manuscript. NG, DM, and RP tested and evaluated the package and suggested several modifications. RP and NG helped with database design. FL gave

mentorship over direction of project including focus on metadata collection and addition of customized fields for user. All authors read and approved the final manuscript.

Acknowledgements

The authors would like to acknowledge financial support from Singapore Ministry of Education Academic Research Fund Tier 3 MOE2013-T3-1-013, and the Singapore Centre for Environmental Life Sciences Engineering (SCELSE), whose research is supported by the National Research Foundation Singapore, Ministry of Education,Nanyang Technological University, and National University of Singapore, under its Research Centre of Excellence Program. The authors would also like to thank Megan Clare for her MetaLIMS development support through use and testing in the SCELSE air microbiome group. Thanks also to Wesley Goi for his suggestions to ease deployment of web applications. Special thanks to Stephan C Schuster for his ongoing support and encouragement.

References

[1] Genologics Clarity LIMS. www.genologics.com/claritylims. Accessed 15 Feb 2016.

[2] Starlims. www.abbottinformatics.com/us/products/lims. Accessed 15 Feb 2016.

[3] Scholtalbers J, Rossler J, Sorn P, de Graaf J, Boisguerin V, Castle J, Sahin U: Galaxy LIMS for next-generation sequencing. Bioinformatics 2013, 29:1233-1234.

[4] SciGenom Labs. www.scigenom.com. Accessed 15 Feb 2016.

[5] MISO Managing Information for Sequencing Operations. http://www.earlham.ac.uk/miso/. Accessed 9 Nov 2016

[6] BIKA LIMS. https://www.bikalims.org/. Accessed 9 Nov 2016

[7] SIERRA LIMS. http://www.bioinformatics.babraham.ac.uk/projects/sierra/ . Accessed 9 Nov 2016

[8] Grimes SM, Ji HP, MendeLIMS: a web-based laboratory information management system for clinical genome sequencing, *BMC Bioinformatics*,2014;27;15:290

[9] Sapio's Laboratory Information Management (LIMS) Solution. www.sapiosciences.com. Accessed 15 Feb 2016.

355 [10] Wetterstrand KA. DNA Sequencing Costs: Data from the NHGRI Genome Sequencing Program (GSP). www.genome.gov/sequencingcosts. Accessed 15 Feb 2016.

357 [11] Hayden EC. Technology: The $1,000 genome. http://www.nature.com/news/technology-the-1-000-genome-1.14901. Accessed 16 May 2016.

359 [12] Amazon Lightsail. www.amazonlightsail.com. Accessed 13 Jan 2017

360 [13] Brady. www.bradyid.com. Accessed 15 Feb 2016

361 [14] Zebra Technologies. www.zebra.com/gb/en/products/printers/desktop.html. Accessed 15 Feb 2016.

362 [15] Acerbi, E., Chénard, C., Miller, D., Gaultier, N. E., Heinle, C. E., Chang, V. W.-C., Uchida, A., Drautz-Moses, D. I., Schuster, S. C. and Lauro, F. M. (2016), Ecological succession of the microbial communities of an air-conditioning cooling coil in the tropics. Indoor Air. doi:10.1111/ina.12306

365 [16] Omics Tools. www.omictools.com/lims-category. Accessed 13 Feb 2017

Figure1

Click here to download Figure Figure1.PNG

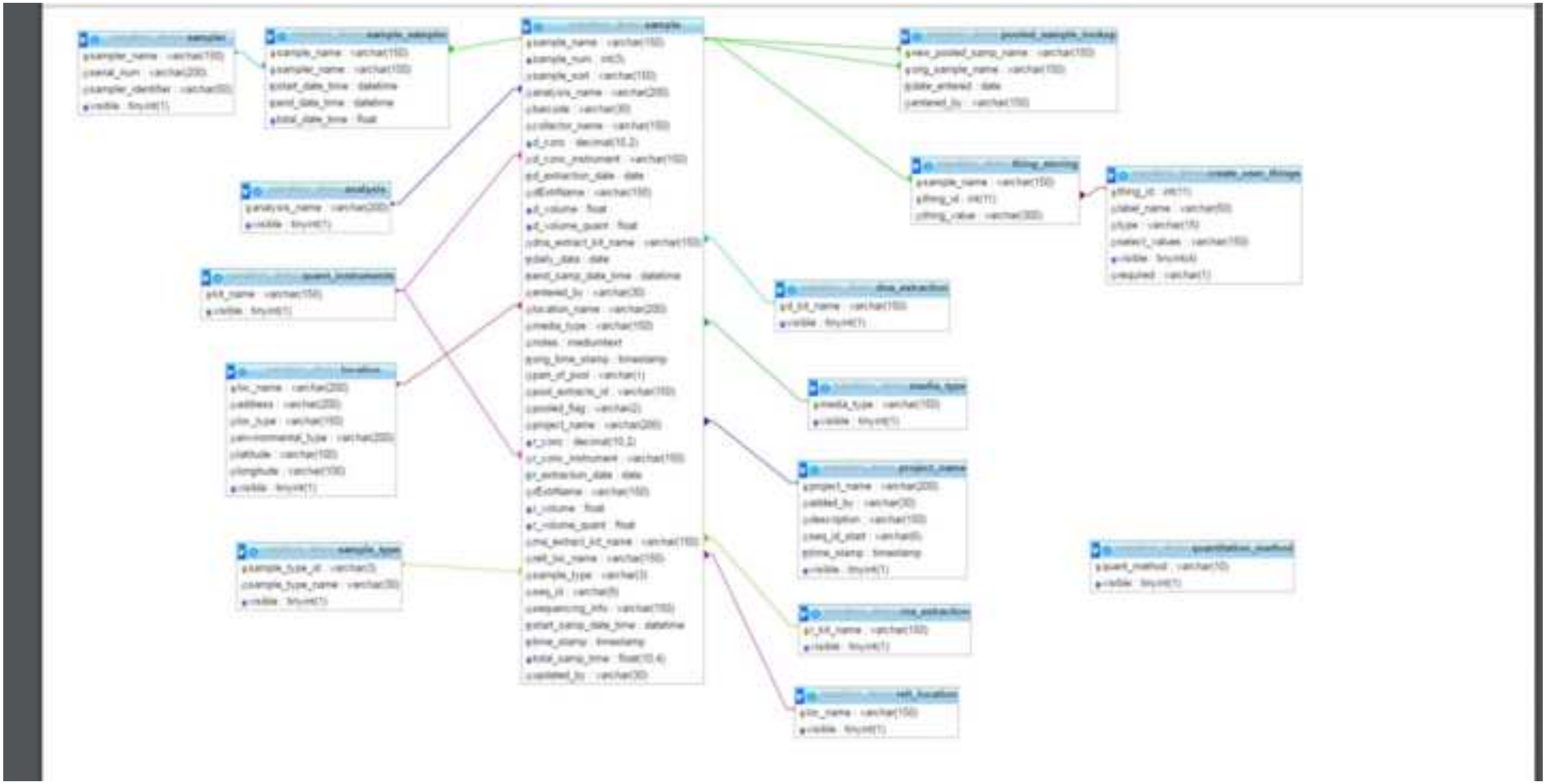

Figure2

Click here to download Figure Figure2.png

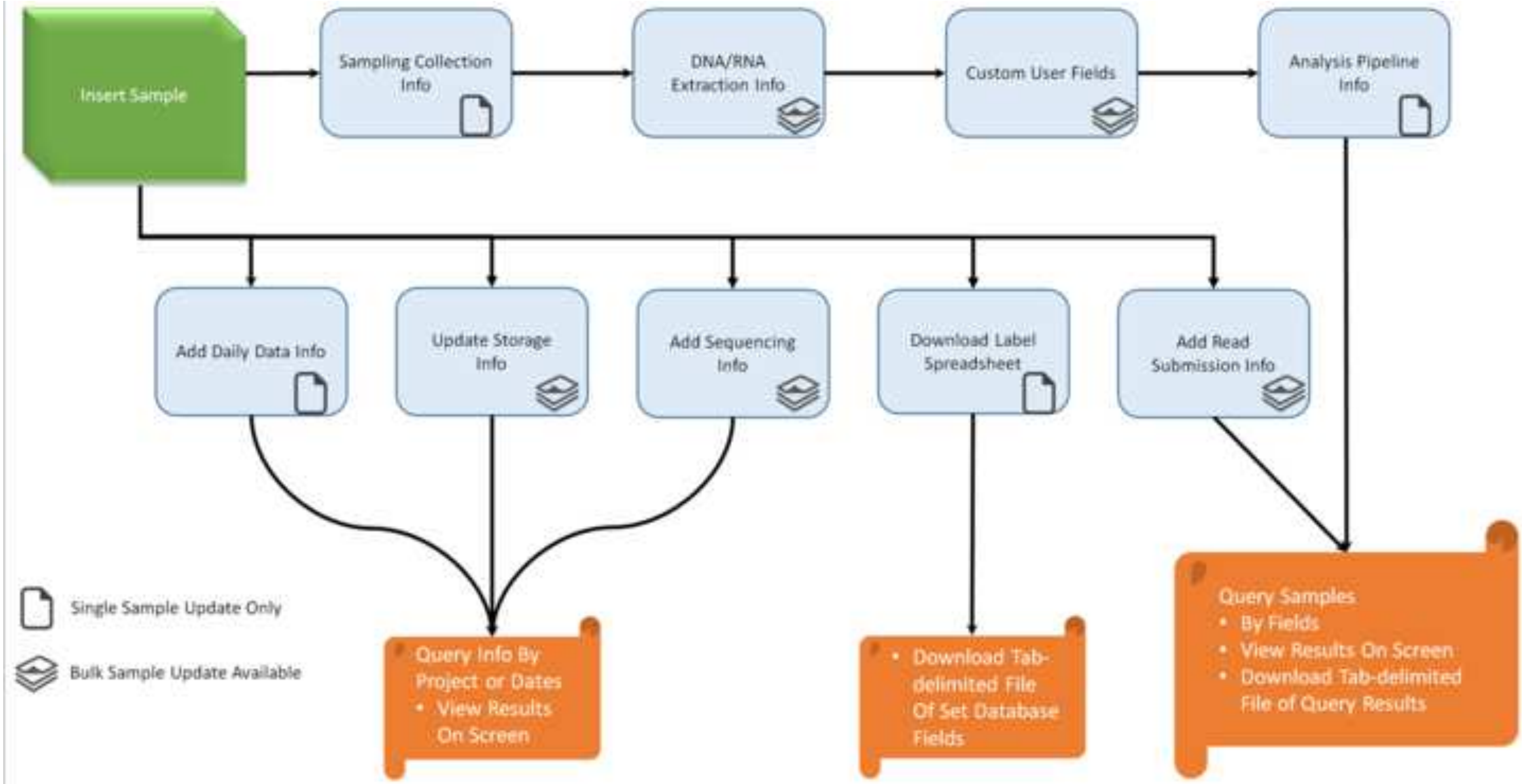

Figure3

Click here to download Figure Figure3.png

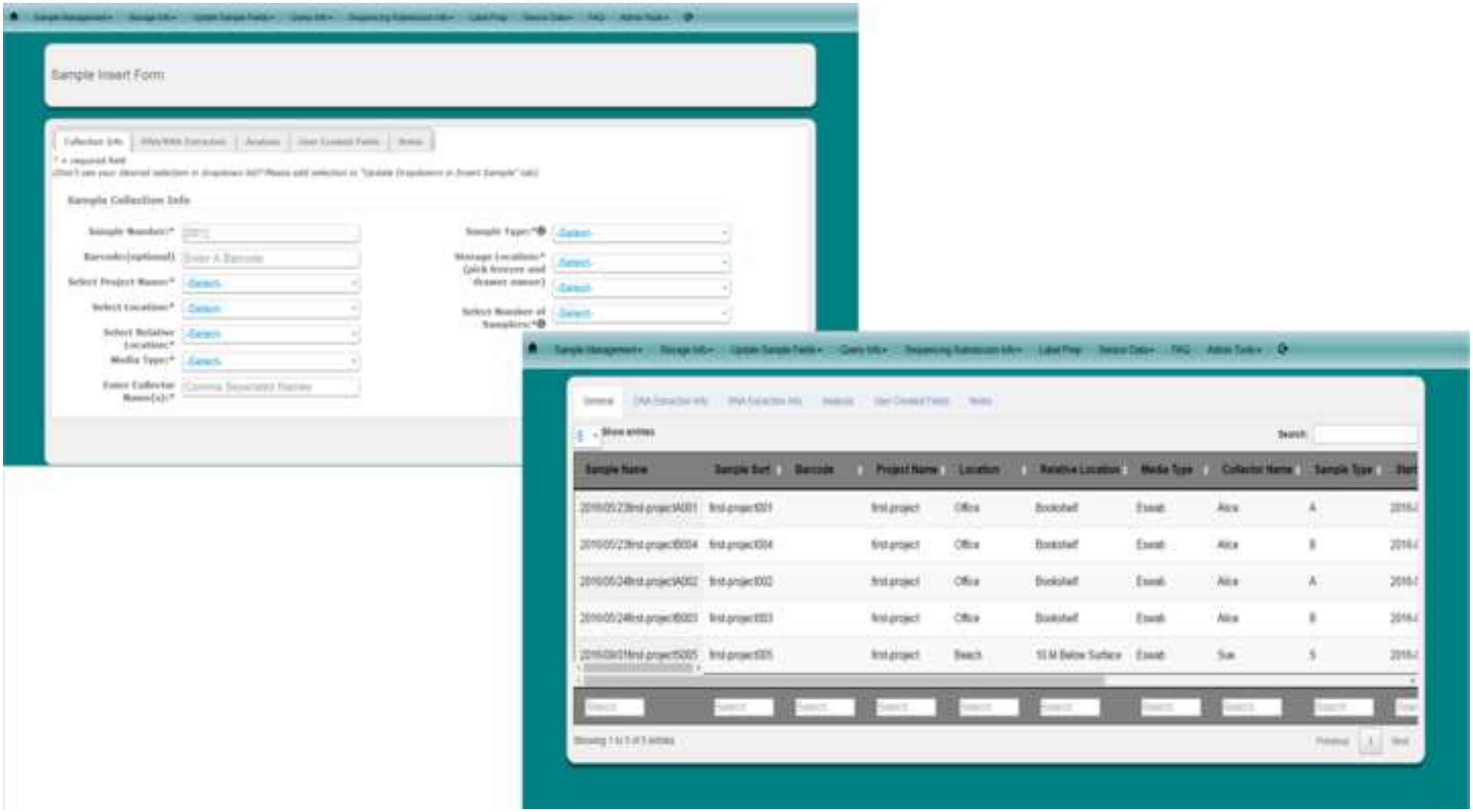

Supplementary Figure1

Click here to access/download
Supplementary Material
Figure1.pdf

Personal Cover
Click here to download Personal Cover metalims_cover_letter_cheinle.pdf

SCELSE
Singapore Centre for Environmental Life Sciences Engineering

NANYANG TECHNOLOGICAL UNIVERSITY

Singapore, February 12, 2017

Editor-in-chief
Dr. Laurie Goodman
GigaScience

Dear Dr. Goodman,

Please find attached the extensively revised manuscript entitled "*MetaLIMS, A Simple Open-Source Laboratory Information Management System for Small Metagenomic Labs*", by Heinle et al. to be considered for resubmission to GigaScience. This manuscript was first submitted on June 23, 2016 under the title "*NanoLIMS, A Simple Open-Source Laboratory Information Management System (LIMS) for Small Sequencing Labs*. NanoLIMS has since been renamed MetaLIMS due to discovery of possible IP conflict.

MetaLIMS, our web deployable laboratory information system, aims to help labs achieve powerful simple sample management, now needed as smaller projects begin to gain access to and produce large datasets. MetaLIMS is uniquely built to store pre-sequencing pipeline metadata for labs which outsource their sequencing and therefore do not need the extra bulk of library preparation and sequencing data associated with many common NGS LIMS. Specifically, MetaLIMS aims to provide flexibility in what is recorded for each sample by offering custom user created fields. It also seeks to alleviate the burden of cost found with current LIMS with this same flexibility. It is our hope that by offering MetaLIMS as both a free and open-source LIMS solution that the community may, if needed, be able to extend and easily build upon MetaLIMS to further fit their needs.

We have fully the addressed concerns by previous reviewers at GigaScience, pertaining to difficulty deploying MetaLIMS and defining its intended beneficiaries. As a result of the review process, we feel that the revised manuscript and the MetaLIMS software are much improved. For a detailed response to each review comment please see details attached.

The paper conforms to the journal's style and format, and has not been published or submitted for publication elsewhere. The manuscript has been seen and approved by all listed authors.

Looking forward to your considered response.

Sincerely,

Cassie Heinle, M.S.
Research Associate

Singapore Centre for Environmental Life Sciences Engineering (SCELSE)
Nanyang Technological University
60 Nanyang Drive, SBS-01N-27
Singapore 637551
E-mail: cheinle@ntu.edu.sg

Response to Original Submission
Click here to download Personal Cover metalims_original_submission_reply_cheinle.pdf

In direct reply to original reviewers concerns:

Reviewer reports:

Reviewer #1: The authors present NanoLIMS, a free and open source LIMS. The authors correctly identify the lack of a low-cost LIMS system as an important limitation for many small labs. I have one serious concern about this particular system however, and that is the installation process. The authors advertise in their abstract that this system is intended for labs that may not have dedicated IT personnel. This means that the system must be easy to set up for biologists who likely have no command line experience. The online install instructions then begin by pointing the user to googling LAMP setup (which inevitably, I think, will be a command line driven process) and then a series of command line sets for setup. I think the authors need to make this system available as a binary for ease of installation (e.g., Anaconda is becoming very popular for binary distribution, and should work for this) or revise their claims about the system to indicate that it will require a system administrator to setup and maintain (e.g., install updates to NanoLIMS or the underlying LAMP stack).

Author response:

> To address the difficulty in deploying MetaLIMS (formerly NanoLIMS), the authors have created a second type of deployment and improved documentation to remove some of the complications associated with web-deployment. Suggestion by authors for the use of web hosted systems removes difficulty for users managing and securing their own web server as well as the task of backing up data. Inclusion of bash scripts to install required prerequisites and MetaLIMS make deployment using this method a 2-3 step process, therefore significantly reducing the aforementioned deployment complexity.

I have several other smaller comments.

First, I recommend a more permissive license than GPL, which prevents integration with other non-GPL licensed software. LGPL and BSD are more permissive choices. A good discussion of this can be found here: http://nipy.org/nipy/faq/johns_bsd_pitch.html#johns-bsd-pitch

----

Author response:

> GPL license not changed - Decision by authors not to change the GPL License with MetaLIMS (formerly NanoLIMS). Current understanding of GPL software license includes that if you use GPL software, your project must also be released under the same license (https://lukasa.co.uk/2012/05/GPL_vs_MIT_Which_License_To_Use/). Due to the inclusion of PhpExcel with is under the GPL license, MetaLIMS is also being released under the GPL license.

Next, I recommend that the authors make a demo video that can be included as a supplementary file. This will be useful for demonstrating what the system can do.

Author response:

Improved documentation without video - There was a split between authors and colleagues on whether to include a demo video. Many felt that they personally did not in general benefit from watching demo videos. Decision was to forgo creation of demo video in lieu of improved documentation on GitHub.

There are currently no releases available on GitHub. The authors should produce official releases on GitHub to support reproducibility.

Author response:

Release of MetaLIMS (formerly NanoLIMS) at GitHub has been created.

Finally, the figures are very blurry, for example Figure 2 is illegible.

Author response:

Figures have been changed. Original Figure 2 no longer exists. Image size and dpi for all images have been set to the formatting standard as outlined in the GigaScience submission guidelines.

Reviewer #2: I, Robert Davey, promise:

- to not hide behind a screen of anonymity
- to be open and honest with you (the authors) at all times
- to be constructive in my criticism
- within the rules given to me by the journal, to assist you in every way I ethically can to get your manuscript published, by providing criticism and praise that is valid and relevant

The technical note outlines "NanoLIMS, A Simple Open-Source Laboratory Information Management System (LIMS) for Small Sequencing Labs", a web-based software tool to aid sequencing labs in sample tracking through lab processes.

The paper is generally clear with no major issues in terms of structure, content, or conventions regarding code availability. The code is freely available on GitHub under the GPLv3, and there are adequate user and developer documentation pages to assist. The GitHub repository has a license.md and README.md.

However, I do have some minor comments:

- There are a much larger number of available LIMS, some very much overlapping with the author's list of NanoLIMS's features. A comprehensive list is here: https://omictools.com/lims-category. Whilst I wouldn't expect a complete review of all existing LIMS to be necessary, I would at least expect the recently updated open-source versions be compared to NanoLIMS. For example, sierra LIMS, MISO (I will note that I am the original developer of MISO), Bika, and MendeLIMS are all open source and are available as sample tracking LIMS from the outset. It would be helpful to understand how the feature set of NanoLIMS compares to those of these tools to enable potential users to understand the benefits of NanoLIMS in their labs.

Author response:

Added comparison of MetaLIMS (formerly NanoLIMS) to recommended open-source LIMS to help define the differences between current LIMS and MetaLIMS (see Table 1).

- NanoLIMS is promoted as a simple and low-maintenance LIMS that is suitable for labs with little or no IT provisioning. Upon reading the developer documentation, it becomes clear that NanoLIMS has no delete functionality available to users within the web interface (e.g. https://github.com/cheinle/NanoLIMS/wiki/NanoLIMS-v1.0-Manual---For-Developers#known-constraints). Instead, a DB admin has to manually delete rows from relevant tables to remove samples/pools/etc. This isn't user friendly, is prone to error, and these limitations should be explcitly mentioned in the manuscript to better inform users of current functionality and proposed future improvements.

Author response:

The decision to not include a delete function was made in order to prevent accidental deletion of samples and to ensure posterity of sample collection. While no delete function currently exists, the users are able to easily edit samples to make corrections in date, project name, and sample number. This limitation is now explicitly stated in user documentation for both users and admins.

- The database schema supplied with the software uses latin1 encoding, rather than UTF-8. Many international characters will be unsupported using this encoding. The authors should strongly consider updating their code and schemas accordingly to allow a broader international user community to use their software fully.

Author response:

Updated tables to charset utf8 and collation type utf8_unicode_ci

----

- It is unclear from the manuscript if NanoLIMS supports the actual submission of read data to public repositories, or simply that users are able to retrospectively add an INSDC accession after submitting the reads manually via a SRA/ENA/DDBJ service. This should be clarified.

Author response:

Clarified in documentation and manuscript that read data submission is only for recording and not for actual submission.

- I am unclear as to why the authors have chosen a 20-field limit (10 text, 10 numeric) for user created information. From the supplied database schema, it seems that this is due to 20 specific rows in a specific table. This could easily be refactored to allow any number of user-created fields of any type (alphanumeric). Is there a reason the authors chose to limit the user fields to this arbitrary amount?

Author response:

Restriction to 20 custom fields removed. MYSQL tables re-designed to store unlimited number of fields.

- Whilst the manuscript does state that NanoLIMS has been used in a local installation and hasn't been tested in a live internet environment, the authors are using dangerously insecure code to generate password reset keys, i.e. rand(). Specific libraries exist to generate keys based on more secure PRNG code, e.g. openssl_random_pseudo_bytes(). This code should be updated.

Author response:

Key generation for reset password token is changed and now uses more secure random_bytes() function (https://github.com/paragonie/random_compat) in lieu of the suggested openssl_random_pseudo_bytes()

- NanoLIMS makes heavy use of XLS templates. To enable wider use, the authors should consider the use of CSV to avoid Excel versioning issues and non Excel users. However, this is not a point that affects my recommendations for acceptance of this manuscript as I realise the relative ubiquity of Excel.

Author response:

File format for downloaded files has been changed to tab-delimited files (.txt) to remove problems with excel versioning and those without access to Excel. Tab delimited files can still be opened and manipulated in Excel as needed by users.

Reviewer #3: NanoLIMS is an open-source laboratory information management system for small sequencing labs. Its claims include: it is free; easy to set up, customize and maintain for users who are not heavily technical; and allows flexibility in the fields that are recorded. Some basic reporting is built in. It is written in PHP and uses a MySQL database, is intended to be deployed on a private network.

Small labs on the cutting edge of research require flexibility for recording sample information and have specific and unique data collection requirements. NanoLIMS claims to be a flexible and general purpose LIMS, but this is not evident in the figures or the NanoLIMS interface. NanoLIMS is set up with default sample fields, transitions and naming schemes. Its conceptual model for how samples are recorded is shown in Figure 3, supplementary figures 2, and 3, but these figures have no text description to accompany them. Some of the terminology is unfamiliar, and so must be explained either in the text or the documentation. The current LIMS is clearly intended for environmental metagenomics, but this information is never shared in the text.
For example, the default fields when adding a sample include "location", "relative location", "collector name", and "samplers". I MUST enter these values in order to proceed. In addition, there are fields for "flow rate" and "height above floor". The "Daily Data" is for measuring the output from a number of sensors at a particular location, which is not something required for an NGS LIMS. The concept of "sampling" seems very tied to sampling from water or air: the "add sampler" page even has a tooltip that says "What is an Air Sampler Identifier?".

Author response:

Scope of MetaLIMS (formerly NanoLIMS) redefined to be specifically for small metagenomics labs prior to input into NGS sequencing pipelines and is now defined as such in the manuscript and in the GitHub wiki.

Fields specific to authors lab, "flow rate" and "height above floor", are removed. "Air Sampler Identifier" is corrected to "Sampler Identifier".

In order to validate the claims of the software itself, I installed and tested NanoLIMS on my own computer.

Free: The code is freely available on Github, and also able to be downloaded as a ZIP if a user cannot use git for whatever reason. The GPL3 license is clear and available in the repository.

Easy to set up, customize, and maintain: The authors claim that it works on Chrome and Firefox, but do not mention Internet Explorer. IE is the preferred browser for many researchers and so NanoLIMS should be tested in the most recent IE version. I had considerable difficulty setting up NanoLIMS on my own computer. I sent the authors a request for assistance via a bug report, and our interactions are in Issue #1 on the Github tracker: https://github.com/cheinle/NanoLIMS/issues/1 The corresponding author has been very responsive, but it took six interchanges to be able to log in to the system. There were several errors in the documentation and at least one SQL error that I found. I would not have been able to debug the application without prior knowledge of debugging MySQL and Apache. As for it being easy to customize, the fields that the admin user can edit are called one thing in the "Update Dropdown" screen and another in the forms, e.g. "Quant Instruments" versus "Instrument/Kit Used to Measure DNA Concentration:". This is not terrible as long as the documentation clearly shows the mapping, but it does not.

Author response:

Not extensively developed on IE - Due to many versions of IE and larger differences in old IE with current browser standards, MetaLIMS (formerly NanoLIMS) has not been extensively developed for use with IE. However, on cursory tests, MetaLIMS works correctly with the new Microsoft Edge .

Secondary installation documentation suggesting use of web-hosting and author created bash scripts, removes many of the troubles encountered by reviewer3 in GitHub issue #1 by setting the correct environment variables and prerequisites needed for correct deployment with minimal need for user input. Additionally, full manual installation documentation used by reviewer3 was updated to call out these needed prerequisites. Specific errors called out by reviewer3 in issue#1 regarding the documentation were corrected.

Field names updated to match naming convention in "Update Drop Down" screen.

Flexible: "Admin users are able to add up to 10 custom entries" (line 185). Why limit the number? If for cosmetic reasons, consider fixing the interface rather than requiring users to modify their entry to fit the arbitrary limit. I was able to add and edit entries into the LIMS without any problems. The one concern that I have about flexibility is that the possible fields are fairly rigorously defined by database tables, and are perhaps specific to the authors' lab or institution. There is a table called "Relt location", for example. There is no documentation as to what these tables mean. They can be hidden using the admin menu if not required.

Author response:

Limitation of number of custom entries is removed. Table names renamed to give clearer meaning to what they are (e.g. "Relt location" updated to "Relative Location")

Overall: Setting up NanoLIMS was difficult and required contacting the authors. The lack of documentation makes customization difficult. Although there are manuals describing how to work with the LIMS, there is no overall explanation of the concepts that underlie NanoLIMS. This information is essential for the small lab starting with a LIMS for the first time. The LIMS is designed for metagenomics sequencing and many screens, fields and default values are specifically for that purpose, but it is never stated in the text. A lab that sequences solid tissue, biobanked samples or blood would need to customize most of the interface, and the documentation is not complete enough easily do so.

NanoLIMS should either become more a more generic LIMS or declare itself as a metagenomic LIMS. Regardless, it requires better documentation to support its claims of flexibility.

Author response:

Scope of MetaLIMS (formerly NanoLIMS) redefined to be specifically for small metagenomics labs prior to input into NGS sequencing pipelines and is now defined as such in the manuscript and in the GitHub wiki.

Figure added to GitHub indicating overall concepts underlying MetaLIMS for users new to LIMS
