# Supplementary material for: MetaLIMS, a simple open-source laboratory information management system for small metagenomic labs
Source: Gigascience. 2017 Apr 18;6(6):1–6. doi: 10.1093/gigascience/gix025 (PMC5449644; doi:10.1093/gigascience/gix025)
Supplement: GIGA-D-17-00035_Revision_1.pdf [file gix025_GIGA-D-17-00035_Revision_1.pdf]

Manuscript
Click here to download Manuscript metalims_manuscript_cheinle.pdf

Title page

MetaLIMS, A Simple Open-Source Laboratory Information Management System for Small Metagenomic Labs

Cassie Elizabeth Heinle (corresponding author)
cheinle@ntu.edu.sg
SCELSE
Nanyang Technological University
60 Nanyang Dr, 637551

Nicolas Paul Eugène Gaultier
GNPEugene@ntu.edu.sg
SCELSE
Nanyang Technological University
60 Nanyang Dr, 637551

Dana Miller
dana.miller@ntu.edu.sg
SCELSE
Nanyang Technological University
60 Nanyang Dr, 637551

Rikky Wenang Purbojati
rpurbojati@ntu.edu.sg
SCELSE

Nanyang Technological University
60 Nanyang Dr, 637551

Federico M. Lauro
FLauro@ntu.edu.sg
SCELSE-NTU
Nanyang Technological University
60 Nanyang Dr, 637551

Abstract

Background

As the cost of sequencing continues to fall, smaller groups increasingly initiate and manage larger sequencing projects and take on the complexity of data storage for high volumes of samples. This has created a need for low-cost laboratory information management systems (LIMS) that contain flexible fields to accommodate the unique nature of individual labs. Many labs do not have a dedicated IT position, so LIMS must also be easy to setup and maintain with minimal technical proficiency.

Findings

MetaLIMS is a free and open source web-based application available via GitHub. The focus of MetaLIMS is to store sample metadata prior to sequencing and analysis pipelines. Initially designed for environmental metagenomics labs, in addition to storing generic sample collection information and DNA/RNA processing information the user can also add fields specific to the users lab. MetaLIMS can also produce a basic sequencing submission form compatible with the proprietary Clarity LIMS system used by some sequencing facilities. To help ease the technical burden associated with web-deployment, MetaLIMS options the use of commercial web-hosting combined with MetaLIMS bash scripts for ease of set-up.

Conclusions

MetaLIMS overcomes key challenges common in LIMS by giving labs access to a low-cost and open source tool that also has the flexibility to meet individual lab needs and an option for easy deployment. By making the web application open source and hosting it on GitHub, we hope to encourage the community to build upon MetaLIMS, making it more robust and tailored to the needs of more researchers.

Keywords

LIMS-customizable-GitHub-open source-web application-sample management-javascript-php-mysql-html

Background

MetaLIMS is a Laboratory Information Management System (LIMS) for powerful but simple sample management. There are many varieties of LIMS at present, ranging from custom built options to out-of-the-box packages [1-9]. These options come in varying degrees of complexity and specificity starting from those which house sample names and metadata like MetaLIMS, to those that track samples through a pipeline and analyze the data [1][3-9]. The need for basic LIMS packages is increasing as sequencing costs drop, which enables smaller labs to initiate and manage larger sample collections [10][11]. The result is a need for data management power that is greater than that of basic laboratory lab notebook and spreadsheets in order to manage samples. However, the combination of high prices, the time and complexity of set-up and maintenance, and the need for flexibility to meet individual labs' needs are often barriers to adopting LIMS systems, especially for smaller projects [1-9]. To help ease these three problems, MetaLIMS is an easy-to-set-up LIMS that is low in cost, open-source, and has the ability to create custom fields for recording sample information. The goal of MetaLIMS is to allow more researchers to more easily and affordably manage their sample information.

Findings

MetaLIMS differs from other open-source LIMS in that its focus is to store sample collection and processing metadata such as DNA extraction details, prior to downstream sequencing and analysis pipelines. While many smaller labs outsource their sequencing or analysis to expertise outside their group, it is advantageous to have a LIMS which can record information detached from these sequencing and analysis pipelines. MetaLIMS was specifically designed for use by microbiology labs using high throughput sequencing for metagenomic analysis. In addition to storing generic sample collection information and DNA/RNA processing information, the user can also add field's specific to the users' lab. MetaLIMS borrows the utility of web hosting services alongside MetaLIMS installation bash scripts to offer an installation process alongside its more advanced installation documentation to ease installation

processes for users with milder computer experience.

Database Description

MetaLIMS functions using a web based interface and was built and tested using XAMPP v3.2.1 and HTML5. MetaLIMS has been deployed on a production server using Apache 2.2.15 , MySQL 5.5.43 (including mysqli module), PHP 5.5.25 on a closed internet network. MetaLIMS can be deployed on any basic LAMPP stack using Apache 2.2.15 , MySQL 5.5.43, PHP 5.5.25 (with mysqli extension) or newer. Figure 1 shows the database schema for MetaLIMS's main tables involved in sample recording. Full database schema can be found in Supplementary Figure 1.

Figure 1. Database schema of MetaLIMS main sample tables. Please see supplementary Figure 1 for full database schema including auxiliary tables

MetaLIMS uses many open source packages such as Php Excel, DataTables, free html and php login templates, jquery libraries, and Creative Commons wallpapers which come packaged with the source code.

Database access and Deployment

MetaLIMS is free and easily downloaded or 'cloned' from the project's public GitHub page (https://github.com/cheinle/MetaLIMS). A GitHub account is not required. Users can access the MetaLIMS installation and user manuals via the MetaLIMS GitHub wiki (https://github.com/cheinle/MetaLIMS/wiki) . MetaLIMS allow users to customize the way that the database is hosted and backed-up and suggested options for new users can be found on the MetaLIMS wiki. Users will be able to implement their own database and web application security in a custom way which is not possible with many commercial LIMS.

Due to the complexity of many LIMS, most laboratories need a dedicated IT person(s) to set-up and maintain their LIMS. In response to this problem, alongside MetaLIMS more advanced installation instructions, MetaLIMS suggests usage of hosted web services and MetaLIMS installation bash scripts to create simple and streamline deployment and maintenance. Installation instructions were created utilizing one web hosting service, Amazon Lightsail. While MetaLIMS does not endorse these services, Lightsail was chosen due to Amazon's free one month trial enabling users to freely try MetaLIMS on a hosted system [12]. MetaLIMS installation bash scripts can also be used for deployment of prerequisite LAMP stack and MetaLIMS application on any machine running Ubuntu 16.04 or Ubuntu 14.04. MetaLIMS bash scripts were tailored towards Ubuntu due to the large community of researchers using Ubuntu. Future work will involve extensibility of these scripts to other Linux and Unix distributions.

User Interface

Sample recording

MetaLIMS was created to allow labs to easily manage their sample metadata by giving all lab members access to easily contribute, edit, and obtain sample information. Figure 2 shows the MetaLIMS workflow for recording and retrieving sample information. MetaLIMS contains fields to store basic sample information such as when samples were collected and what type of sampling collection method was used. It can also record DNA and RNA extraction information such as the kit used for extraction, which person performed the extraction, and the concentration and volume of extracted samples. MetaLIMS can store sample storage information for other downstream events such as sequencing submission information and which analysis pipeline was used.

Figure 2. Sample recording workflow in MetaLIMS

Users can access sample information one record at a time using the 'Update Sample' function. Here the user can get a detailed view of a particular entry and make any edits or additions as necessary.

Users can also view information for batches of samples by using the ‘Query Info’ function to view samples by date or by specific sample collection or processing criteria. Users will be able to view their sample records on the screen or download selected records as a tab-delimited document for further exploration and data manipulation. Figure 3 shows the sample input form and output for sample queries for MetaLIMS.

Figure 3. MetaLIMS sample input

Custom recording

Custom fields may be created by the admin user for users to enter additional sample information unique to an individual lab or project. Admin users are able to add custom text entries (as either free text or drop down boxes) as well as numeric entries. Admin users will be able to indicate which custom entries should be required entries for each sample. These new custom fields appear in the tab ‘User Created Fields’ on both the sample entry and sample update forms.

MetaLIMS can also record daily averages of any user-specified data under the ‘Sensor Data’ feature. This allows for custom recording of any sensor data by day and location. For example this would allow a user to store measurements as metadata for a set of samples, such as temperature, humidity, or light intensity.

Using these two functions would allow the lab to track and view any desired information that can be measured for either individual samples or a batch of samples.

Sequencing sample sheet

MetaLIMS was originally built to generate output of a sequencing submission form that is ready for downstream entry into a Genologics Clarity LIMS Gold pipeline but has been adapted to output the sample submission form for generic Clarity LIMS sample sheet used in the Clarity LIMS Silver and Run Manager versions [1]. Because of the variety of types of sequencing submission forms used by different

sequencing facilities, additional customization of MetaLIMS by users with more advanced technical skills may be required to generate sequencing submission spreadsheets in the format required by a specific sequencing facility.

Data submission

For posterity, researchers who upload sequencing data to public databases can use the read submission function to keep a record of if sequencing files associated with a specific sample or set of samples have been submitted to repositories such as DDBJ, Genbank,or ENA. This does not create the submission, but stores the information as record keeping for the user. Users can enter the read submission name, date submitted, and type of experiment the sample was included in.

Labels

MetaLIMS gives the user ability to print out labels, ensuring consistent sample naming and labeling within a lab. This helps prevent problems such as illegible or smeared hand-writing, confusing date formats, and vague or redundant sample naming. MetaLIMS allows the user to print out labels containing sample names, sampling date and time, project name, sample type, and sample number, thus making the tube labeling unambiguous.

MetaLIMS can work with common desktop label printers for researchers looking to print labels. This label function can generate a form of QR codes for either sample names or other sample information for barcoding if a ‘barcode’ field is populated for these samples. Alternatively, a tab-delimited file can be downloaded for easy uploading into to common label making software. These common label makers allow users to connect to a ‘database’ such as an excel worksheet, comma-separated text file, or tab-delimited file for text and barcode generation [13][14].

Database application

MetaLIMS is intended as a sample management solution for smaller labs as the responsibility of creating and storing larger amounts of data comes to smaller research groups. MetaLIMS is currently in use by the Air Microbiome group with the Singapore Centre for Environmental Life Sciences Engineering to house sample information from sample collection as well as archiving information on downstream processes performed such as data analysis, sequencing, and read submission to public databases [15]. While there is increasing growth in the number of LIMS being created to try and fill the unique needs of various labs, Table 1 shows a comparison of MetaLIMS to 4 popular open-source LIMS, MISO LIMS [5], BIKA-LIMS [6] ,SIERRA LIMS [7], and MendeLIMS [8] to help define MetaLIMS for user usage.

MetaLIMS differs in comparison to other LIMS in that unlike many LIMS which were created to store and track samples through NGS sequencing pipelines, such as MISO, SIERRA,BIKA-LIMS, or specific for medical use such as MendeLIMS or BIKA-HEALTH, MetaLIMS is defined specifically for use of storing sample meta-data prior to high throughput sequencing pipelines and analysis. While some LIMS offer the flexibility of adding custom fields or custom population of dropdown fields, MetaLIMS offers this capability without the extra bulk of storing downstream sequencing library prep and machine metrics which may not be needed by small labs which do not do their own sequencing. Lastly, while all LIMS compared grant the fluid adaptability of open-source software, many still require extensive Unix or command-line interface knowledge to deploy.

LIMS Software	Miso LIMS	Bika LIMS	Sierra LIMS	MendeLIMS	MetaLIMS
**For NGS sequencing**	Yes	Not specific	Yes	Yes	No
**For medical sample**	Not specific	Not specific	Not specific	Yes	No
**For sample metadata**	Pre-defined fields and free-form "notes". Sample type configuration for advanced users	Allows sample types	Only sequencing metadata	Yes, for clinical samples	Yes
**Input sample types**	Extracted DNA	Not specific	Extracted DNA/RNA	Clinical samples	Environmental samples
**Customizable - User can create fields**	Can add sequencers	Yes	No	Can populate some configurable fields and dropdowns	Yes
**Web based**	Yes	No	Yes	Yes	Yes
**Software**	JDK7, Tomcat 8, MySQL 5, Flyway, Maven, git	Python	Perl	Javascript, Ruby	PHP
**Database**	MySQL	ZODB (expected PostgreSQL intergration)	MySQL	MySQL, PostgreSQL, or SQLLite	MySQL
**Computer skills [16]**	Advanced	Medium	Basic	Basic	Basic
**Website**	http://tgac.github.io/miso-lims/	https://github.com/bikalabs/bika.lims	http://www.bioinformatics.babraham.ac.uk/projects/sierra/	http://dna-discovery.stanford.edu/software/mendelims/	https://github.com/cheinle/MetaLIMS/wiki

Table 1. Comparison of MetaLIMS to popular open-source LIMS

Conclusions

The decrease in cost of sequencing has led to a subsequent increase in the initiation and management of large data collection by small labs. This increase in the influx in data generated by such projects creates a need for more powerful sample management tools than traditional lab notebooks and spreadsheets. MetaLIMS is able to offer labs easy sharing and access of recorded sample information

across lab members. MetaLIMS is a unique solution which is free and customizable for small metagenomic labs which wish to store metagenomic sample collection and processing information but do not need the extra bulk of recording NGS sequencing or analysis data, which is common in many NGS LIMS. MetaLIMS has demonstrated it overcomes key challenges often associated with LIMS by being free of cost and open source and having customizable sample specific fields to add flexibility to meet the unique needs of different labs. By building MetaLIMS on a common web platform and offering a solution for easy deployment through web-hosting, the complexity of deploying and managing a web application becomes minimal and MetaLIMS becomes easy to set-up and maintain. It is our further desire that making the web application open source and hosting it on GitHub that it will encourage the community to both utilize and build upon MetaLIMS allowing it to become more robust and tailored towards the community's growing needs.

Availability of supporting source code and requirements

Use of MetaLIMS, its data, and source code https://github.com/cheinle/MetaLIMS are unrestricted for use by academic and commercial researchers.

- Project name: MetaLIMS, A Simple Open-Source Laboratory Information Management System for Small Metagenomic Labs
- Project home page: https://github.com/cheinle/MetaLIMS
- Operating system(s): Deployment – Linux, Access- Platform independent
- Programming language: PHP 5.5.25 (including mysqli module)
- Other requirements: Chrome (Version 47.0.2526.111) or Firefox (43.0.4) (preferred), Apache 2.2.15, 266 HTML5, MySQL 5.5.43 (no STRICT_TRANS_TABLES mode)
- License: MetaLIMS is released under the GNU General Public License
- Any restrictions to use by non-academics: None

Declarations

List of abbreviations

AMI: Amazon Machine Image

AWS: Amazon Web Services

LIMS: Laboratory Information Management System

VPS: Virtual Private Server

Ethics approval and consent to participate

Note applicable

Consent for publication

Note applicable

Competing interests

The authors declare that they have no competing interests.

Funding

Singapore Ministry of Education Academic Research Fund Tier 3 MOE2013-T3-1-013, and the Singapore Centre for Environmental Life Sciences Engineering (SCELSE), whose research is supported by the National Research Foundation Singapore, Ministry of Education,Nanyang Technological University, and National University of Singapore, under its Research Centre of Excellence Program.

Authors' contributions

CH implemented the package and wrote the manuscript. NG, DM, and RP tested and evaluated the package and suggested several modifications including feature requests and user interface

improvements. RP and NG helped with database design. FL gave mentorship over direction of project including focus on metadata collection and addition of customized fields for user. All authors read and approved the final manuscript.

Acknowledgements

The authors would like to acknowledge financial support from Singapore Ministry of Education Academic Research Fund Tier 3 MOE2013-T3-1-013, and the Singapore Centre for Environmental Life Sciences Engineering (SCELSE), whose research is supported by the National Research Foundation Singapore, Ministry of Education, Nanyang Technological University, and National University of Singapore, under its Research Centre of Excellence Program. The authors would also like to thank Megan Clare for her MetaLIMS development support through use and testing in the SCELSE air microbiome group. Thanks also to Wesley Goi for his advice to ease and troubleshoot deployment of web applications. Special thanks to Stephan C. Schuster for his ongoing support and encouragement.

References

[1] Genologics Clarity LIMS. www.genologics.com/claritylims. Accessed 15 Feb 2016.

[2] Starlims. www.abbottinformatics.com/us/products/lims. Accessed 15 Feb 2016.

[3] Scholtalbers J, Rossler J, Sorn P, de Graaf J, Boisguerin V, Castle J, Sahin U: Galaxy LIMS for next-generation sequencing. Bioinformatics 2013, 29:1233-1234.

[4] SciGenom Labs. www.scigenom.com. Accessed 15 Feb 2016.

[5] MISO Managing Information for Sequencing Operations. http://www.earlham.ac.uk/miso/. Accessed 9 Nov 2016

[6] BIKA LIMS. https://www.bikalims.org/. Accessed 9 Nov 2016

[7] SIERRA LIMS. http://www.bioinformatics.babraham.ac.uk/projects/sierra/ . Accessed 9 Nov 2016

[8] Grimes SM, Ji HP, MendeLIMS: a web-based laboratory information management system for clinical genome sequencing, *BMC Bioinformatics*,2014;27;15:290

[9] Sapio's Laboratory Information Management (LIMS) Solution. www.sapiosciences.com. Accessed 15

Feb 2016.

[10] Wetterstrand KA. DNA Sequencing Costs: Data from the NHGRI Genome Sequencing Program (GSP). www.genome.gov/sequencingcosts. Accessed 15 Feb 2016.

[11] Hayden EC. Technology: The $1,000 genome. http://www.nature.com/news/technology-the-1-000-genome-1.14901. Accessed 16 May 2016.

[12] Amazon Lightsail. www.amazonlightsail.com. Accessed 13 Jan 2017

[13] Brady. www.bradyid.com. Accessed 15 Feb 2016

[14] Zebra Technologies. www.zebra.com/gb/en/products/printers/desktop.html. Accessed 15 Feb 2016.

[15] Acerbi, E., Chénard, C., Miller, D., Gaultier, N. E., Heinle, C. E., Chang, V. W.-C., Uchida, A., Drautz-Moses, D. I., Schuster, S. C. and Lauro, F. M. (2016), Ecological succession of the microbial communities of an air-conditioning cooling coil in the tropics. Indoor Air. doi:10.1111/ina.12306

[16] Omics Tools. www.omictools.com/lims-category. Accessed 13 Feb 2017

Figure1

Click here to download Figure Figure1.PNG

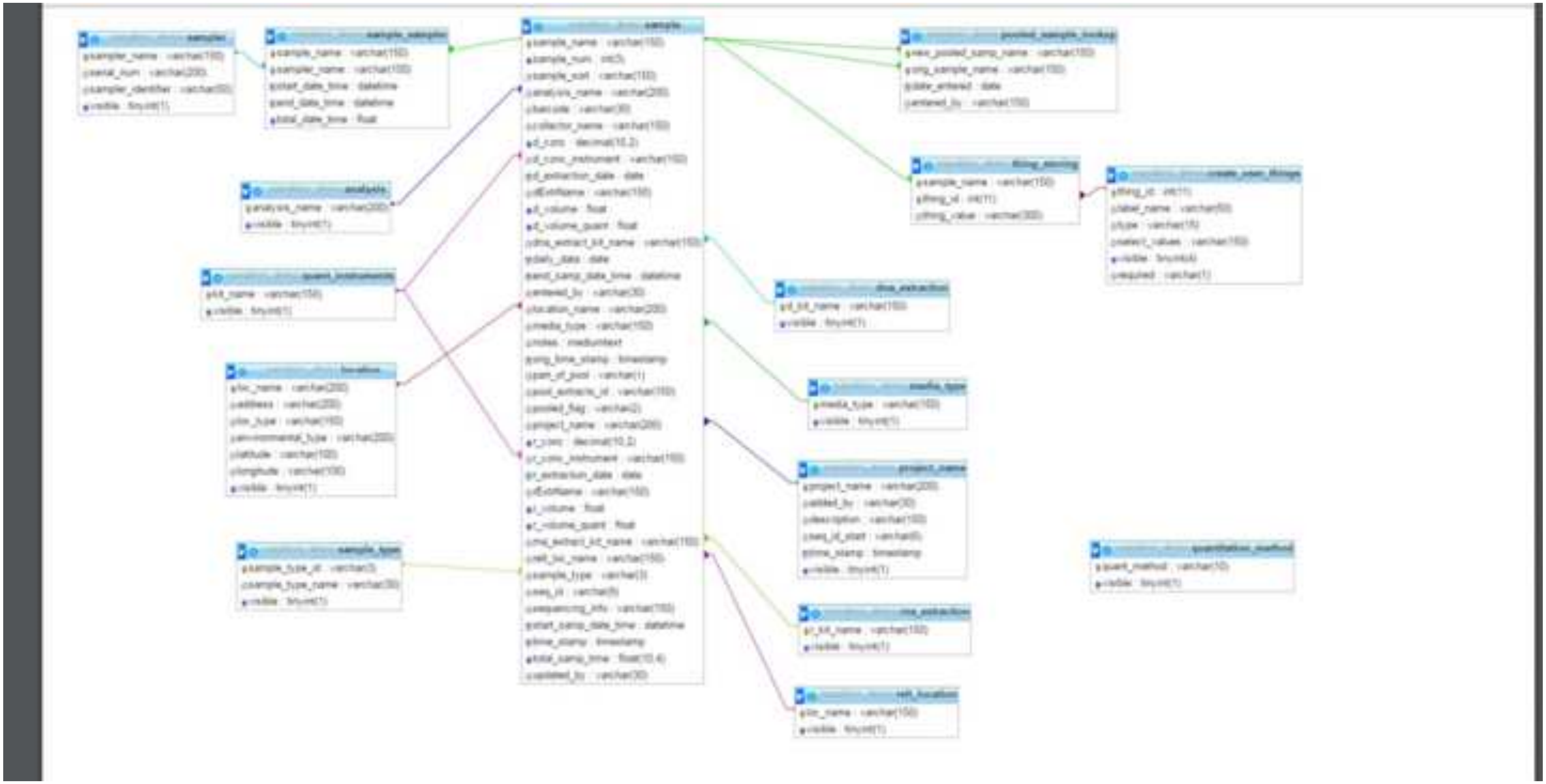

Figure2

Click here to download Figure Figure2.png

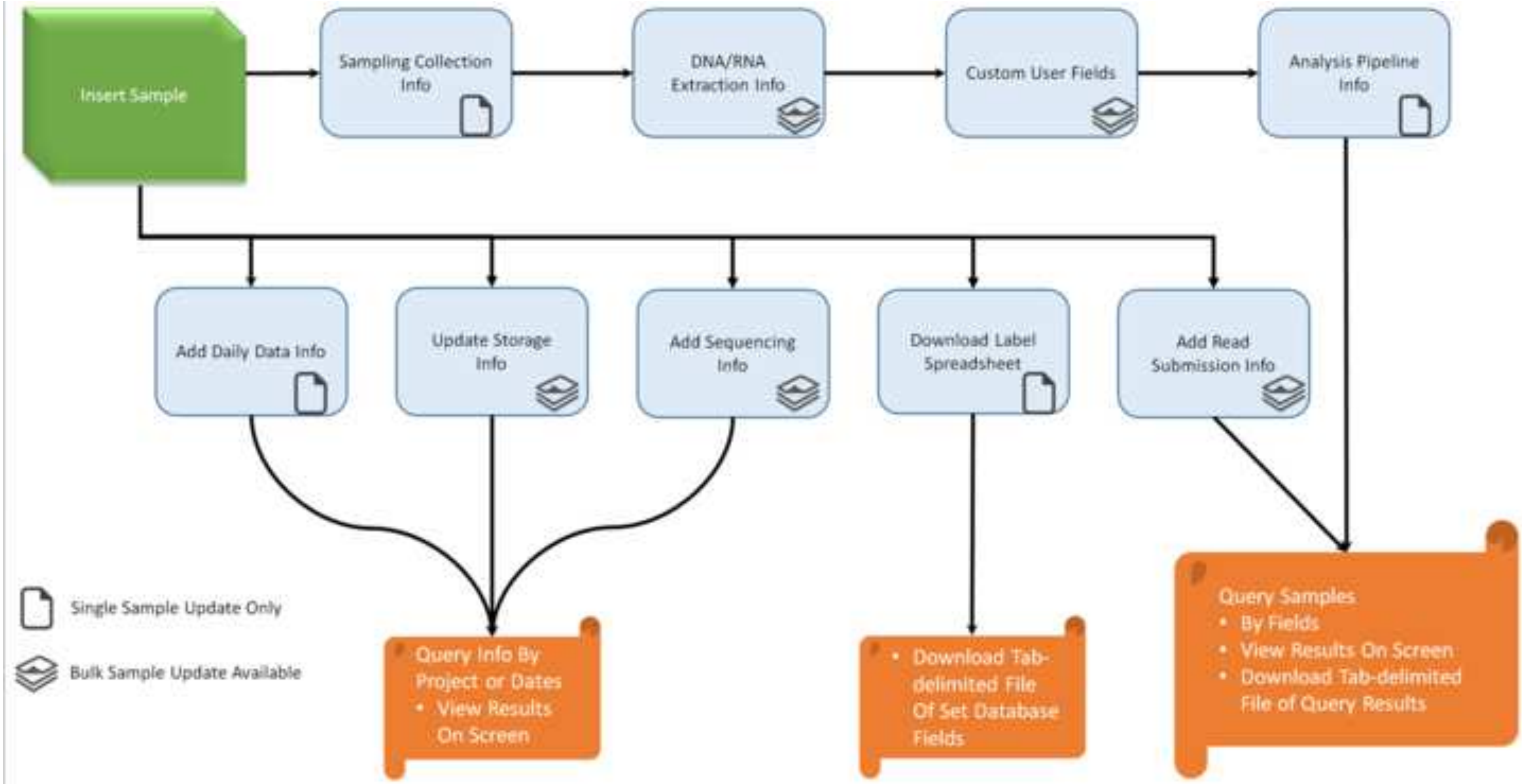

Figure3

Click here to download Figure Figure3.png

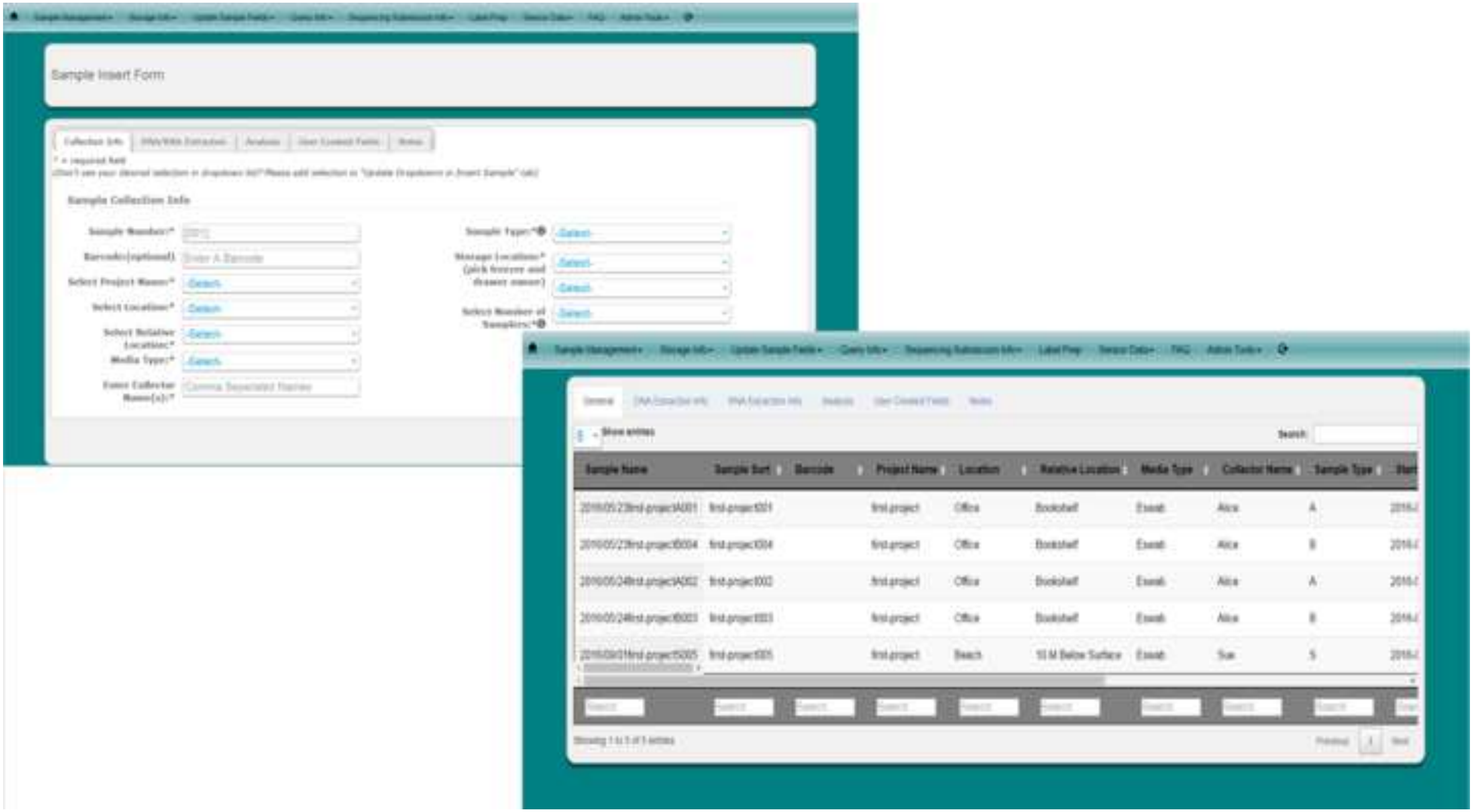

Supplementary Figure1

Click here to access/download
Supplementary Material
Figure1.pdf

Personal Cover

SCELSE
Singapore Centre for Environmental Life Sciences Engineering

Click here to download Personal Cover
metalims_cover_letter_cheinle.pdf

NANYANG TECHNOLOGICAL UNIVERSITY

Singapore, March 14, 2017

Executive Editor
Dr. Scott Edmunds
GigaScience

Dear Dr. Edmunds,

Thank you and the re-reviewer for the time and effort to review our manuscript. Please find attached the manuscript entitled "*MetaLIMS, A Simple Open-Source Laboratory Information Management System for Small Metagenomic Labs*", by Heinle et al. to be considered for resubmission with minor revision to GigaScience. All reviewer suggestions have been addressed and a detailed response to each review comment has been included as intended in the online submission steps. Corrections to author name spelling has also been made.

The paper conforms to the journal's style and format, and has not been published or submitted for publication elsewhere. The manuscript has been seen and approved by all listed authors.

Please let us know how we may best answer any further questions.

Sincerely,

Cassie Heinle, M.S.
Research Associate

Singapore Centre for Environmental Life Sciences Engineering (SCELSE)
Nanyang Technological University
60 Nanyang Drive, SBS-01N-27
Singapore 637551
E-mail: cheinle@ntu.edu.sg
