# Supplementary material for: MetaLIMS, a simple open-source laboratory information management system for small metagenomic labs
Source: Gigascience. 2017 Apr 18;6(6):1–6. doi: 10.1093/gigascience/gix025 (PMC5449644; doi:10.1093/gigascience/gix025)
Supplement: Supplemental material [file gix025_Supp.pdf]

Read Submission

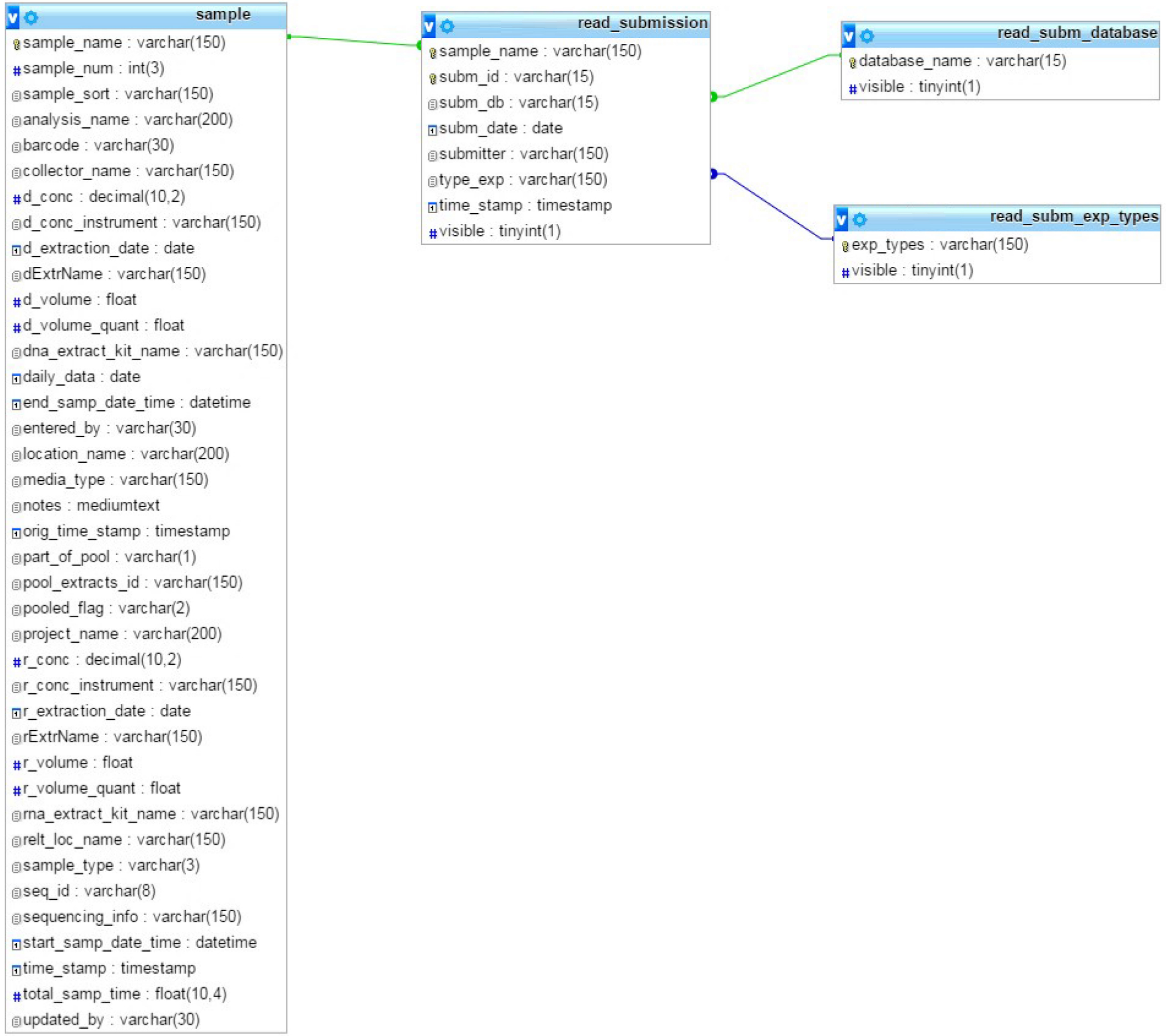

Sample Information

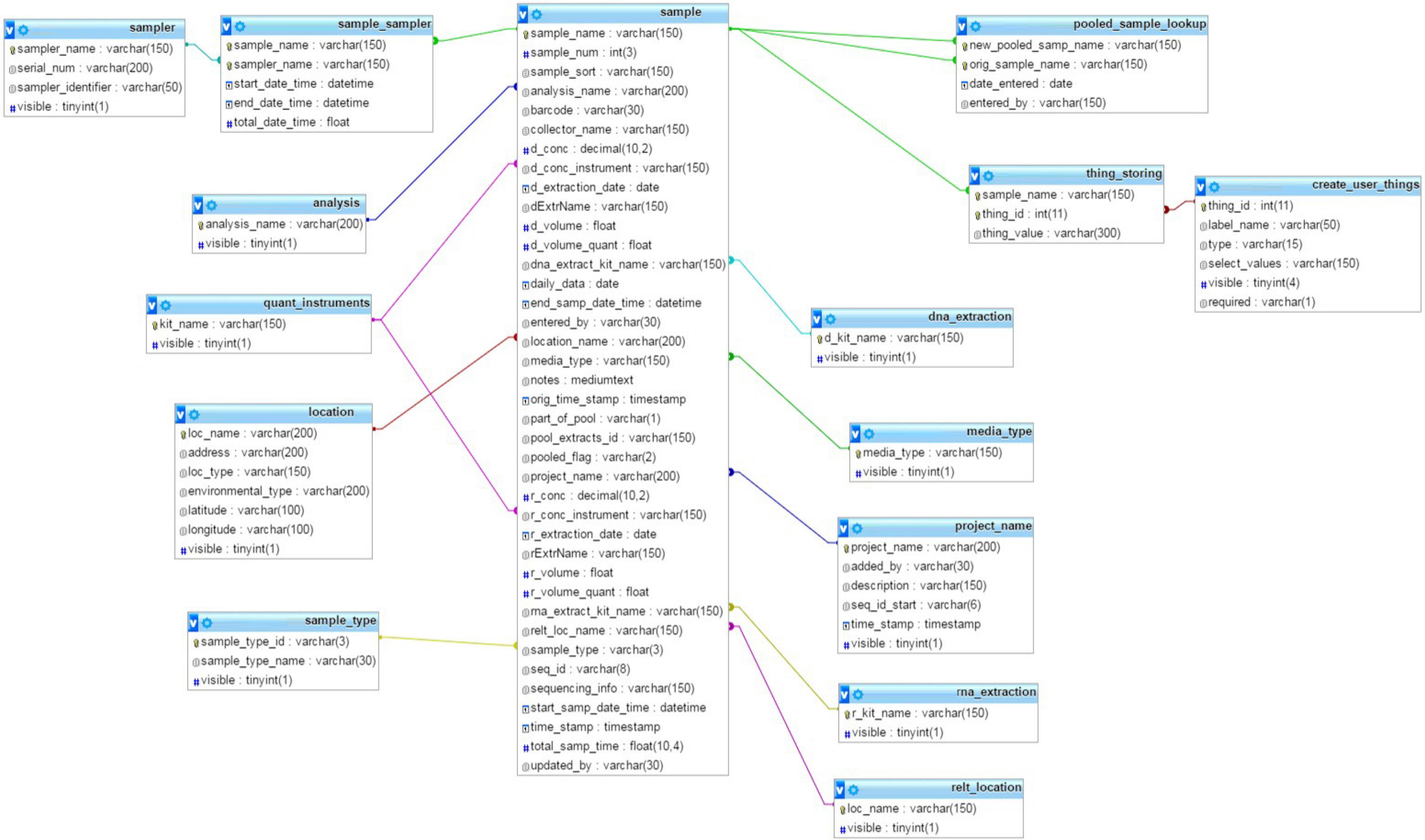

Sample Storage

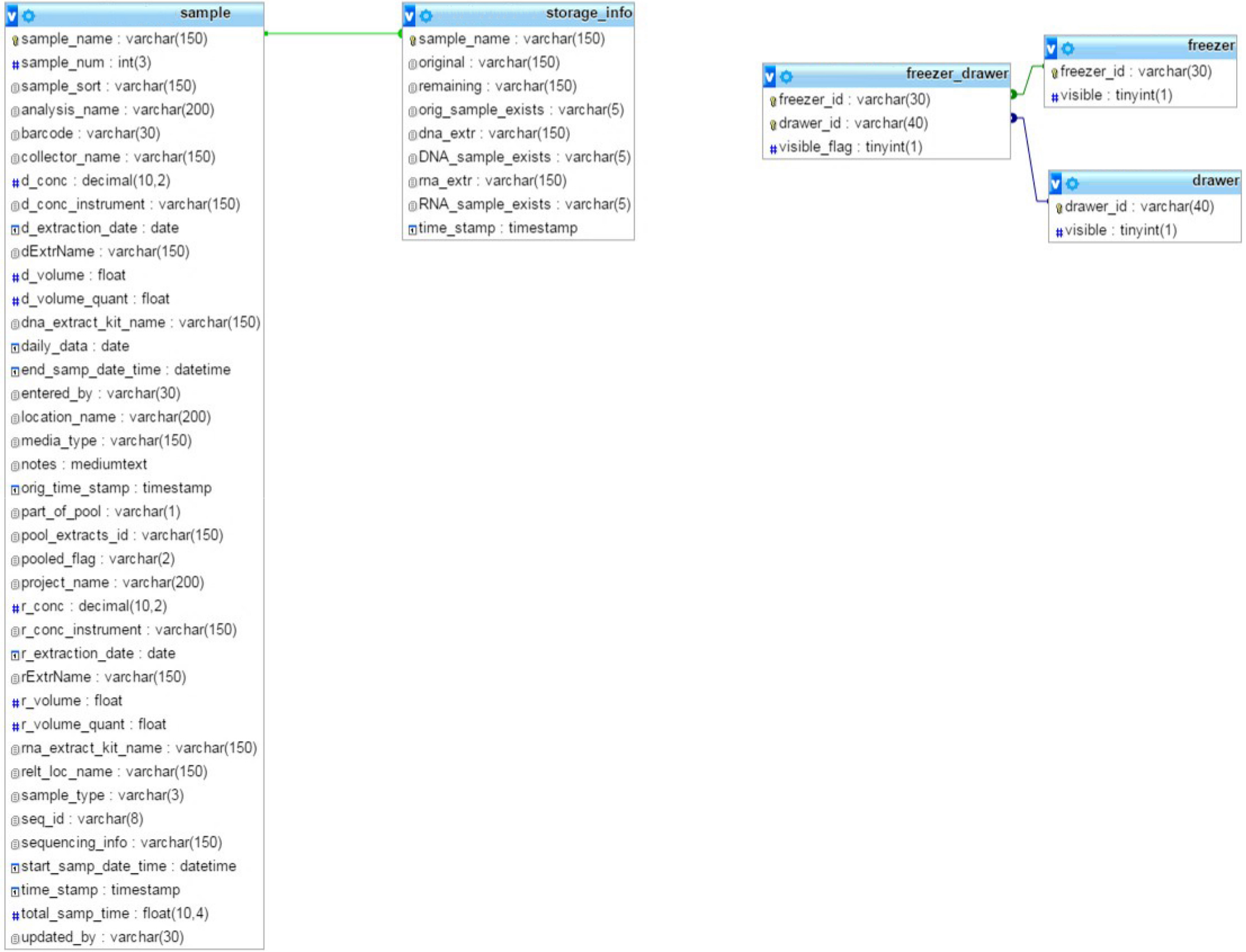

Sensor Data

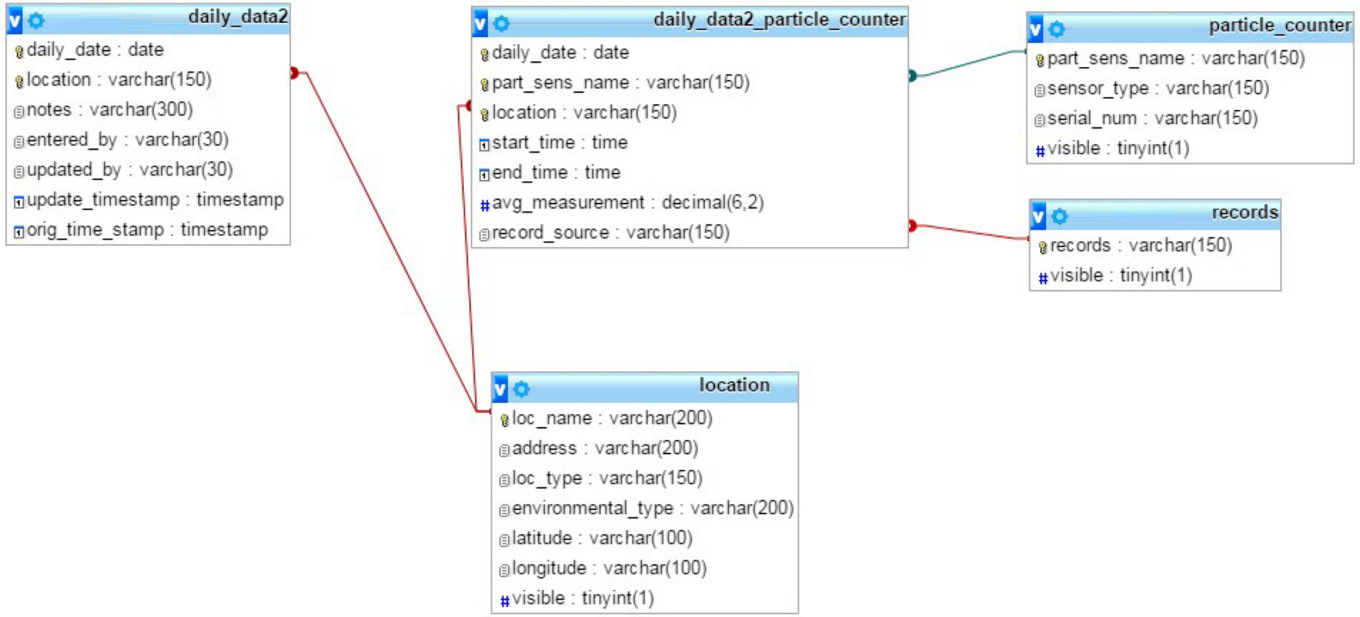

daily_data2
daily_date : date
location : varchar(150)
notes : varchar(300)
entered_by : varchar(30)
updated_by : varchar(30)
update_timestamp : timestamp
orig_time_stamp : timestamp
daily_data2_particle_counter
daily_date : date
part_sens_name : varchar(150)
location : varchar(150)
start_time : time
end_time : time
avg_measurement : decimal(6,2)
record_source : varchar(150)
particle_counter
part_sens_name : varchar(150)
sensor_type : varchar(150)
serial_num : varchar(150)
visible : tinyint(1)
records
records : varchar(150)
visible : tinyint(1)
location
loc_name : varchar(200)
address : varchar(200)
loc_type : varchar(150)
environmental_type : varchar(200)
latitude : varchar(100)
longitude : varchar(100)
visible : tinyint(1)

Sequencing Information

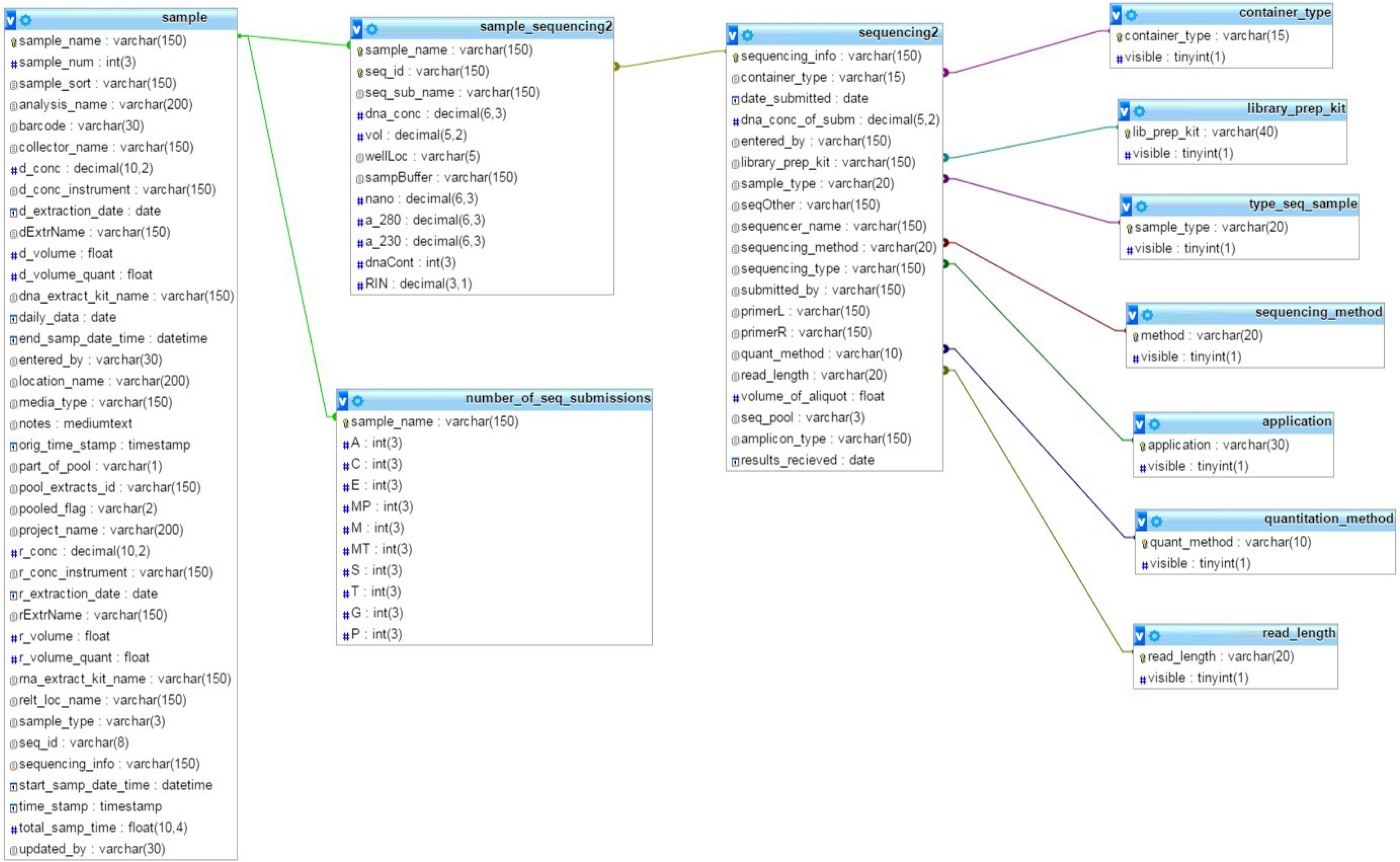

User Created Sample Fields

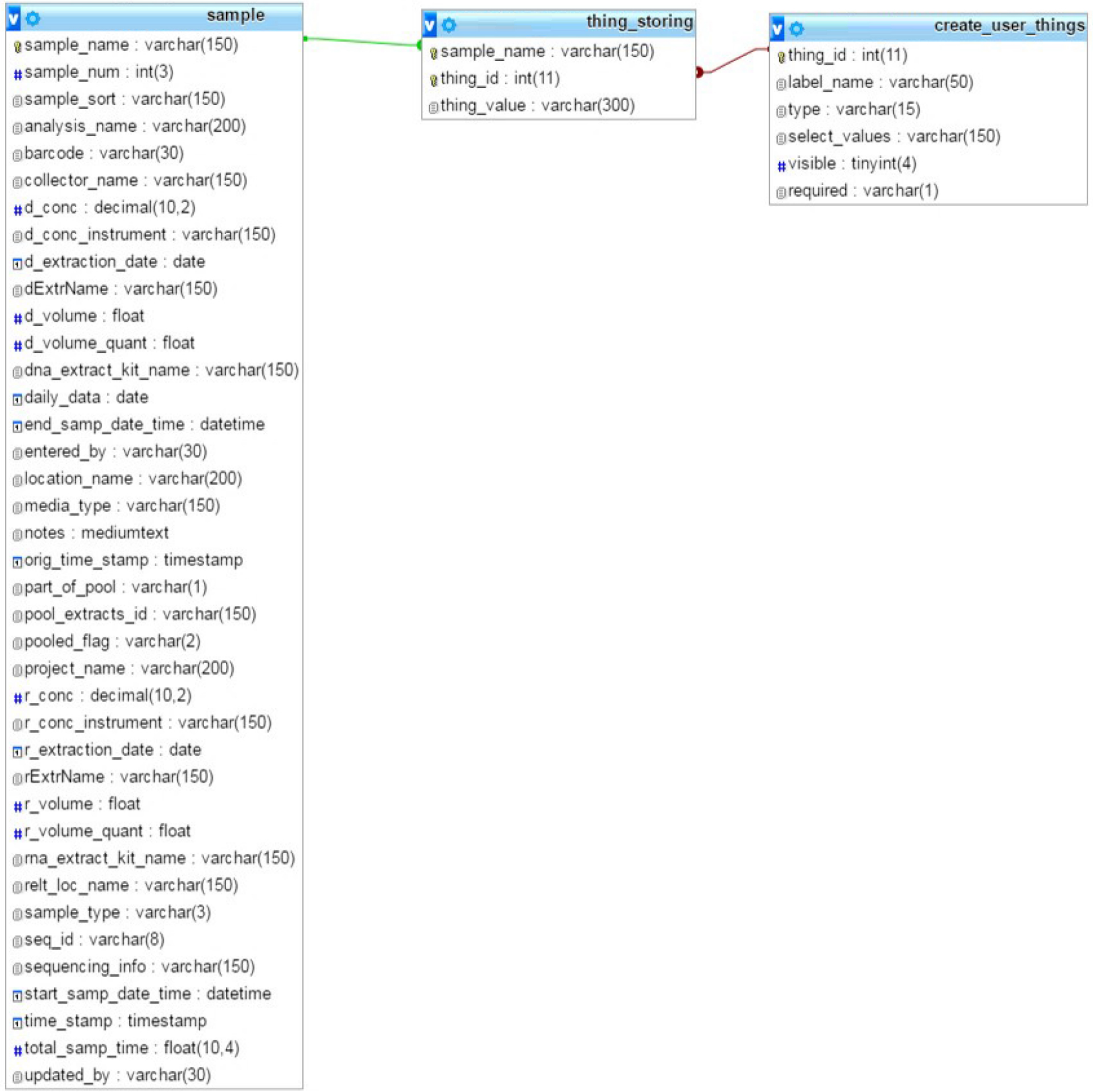

Users

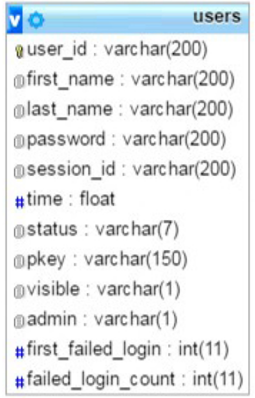
users
user_id : varchar(200)
first_name : varchar(200)
last_name : varchar(200)
password : varchar(200)
session_id : varchar(200)
time : float
status : varchar(7)
pkey : varchar(150)
visible : varchar(1)
admin : varchar(1)
first_failed_login : int(11)
failed_login_count : int(11)
