# Supplementary material for: MetaLIMS, a simple open-source laboratory information management system for small metagenomic labs
Source: Gigascience. 2017 Apr 18;6(6):1–6. doi: 10.1093/gigascience/gix025 (PMC5449644; doi:10.1093/gigascience/gix025)
Supplement: Response_to_reviewer_comments_Original_Submission.pdf [file gix025_Response_to_reviewer_comments_Original_Submission.pdf]

Reviewer reports:
Reviewer #1: MetaLIMS is a lightweight, easy-to-install LIMS system for smaller labs and facilities performing metagenomics studies. It claims to be easy to use and install and simple to configure. I reviewed the first version of this manuscript and found several concerns.

The revised manuscript has addressed all concerns to my satisfaction. MetaLIMS caters to metagenomics labs, an under-represented group in this field. The addition of allowing installation and use of AWS Lightsail as a hosting environment, as well as the overall improvements in documentation, installation, and clarity greatly support the aforementioned claims. It plays nicely with several versions of Genologics LIMS, the current commercial leader, and therefore MetaLIMS does not 'lock' users into it like other LIMS systems do. The authors also include a complete database ER diagram, which was appreciated (possibly not necessary for the publication itself, but definitely should feature in documentation).

The following suggestions are speaking from a slightly biased perspective as one of the developers on MISO LIMS, in table 1. for MISO LIMS, sample metadata can be added at any time, but is limited to pre-defined fields and free-form "notes"; input sample types can be configured through advanced computer skills to include any type of sample (for OICR this starts at 'Tissue'); for software, Eclipse is a development environment, not a deployment software; finally, the correct website is http://tgac.github.io/miso-lims/ .

Author response:

The authors have edited the information in Table 1 for MISO LIMS to correct information as suggested by reviewer-

-Sample metadata has been updated to 'Pre-defined fields and free-form "notes”. Sample type configuration for advanced users'
-Eclipse has been removed for software
-Website for MISO LIMS has been corrected with the given website (http://tgac.github.io/miso-lims/)
