# Supplementary material for: MetaLIMS, a simple open-source laboratory information management system for small metagenomic labs
Source: Gigascience. 2017 Apr 18;6(6):1–6. doi: 10.1093/gigascience/gix025 (PMC5449644; doi:10.1093/gigascience/gix025)
Supplement: Response_to_reviewer_comments_Revision_1.pdf [file gix025_Response_to_reviewer_comments_Revision_1.pdf]

Please add an "Availability of supporting data" section just before the "Availability and requirements". I would suggest something like this:

Availability of supporting data
Snapshots of the supporting code are available from the GigaScience GigaDB repository [17].

Then cite in the references the following:

17. Heinle, C, E; Gaultier, N, P; Miller, D; Purbojati, R, W; Lauro, F, M (2017): Software for "MetaLIMS, A Simple Open-Source Laboratory Information Management System for Small Metagenomic Labs" GigaScience Database. http://dx.doi.org/10.5524/100293

Author response: The additional "Availability of supporting data" section has been added, including the appropriate citation as requested
