# Supplementary material for: MetaLIMS, a simple open-source laboratory information management system for small metagenomic labs
Source: Gigascience. 2017 Apr 18;6(6):1–6. doi: 10.1093/gigascience/gix025 (PMC5449644; doi:10.1093/gigascience/gix025)
Supplement: Reviewer_1_Report_(Original_Submission).pdf [file gix025_Reviewer_1_Report_(Original_Submission).pdf]

Reviewer Report

Title: "MetaLIMS, A Simple Open-Source Laboratory Information Management System for Small Metagenomic Labs"

Version: Original Submission **Date:** 3/6/2017

Reviewer name: Morgan Taschuk

Reviewer Comments to Author:

MetaLIMS is a lightweight, easy-to-install LIMS system for smaller labs and facilities performing metagenomics studies. It claims to be easy to use and install and simple to configure. I reviewed the first version of this manuscript and found several concerns.

The revised manuscript has addressed all concerns to my satisfaction. MetaLIMS caters to metagenomics labs, an under-represented group in this field. The addition of allowing installation and use of AWS Lightsail as a hosting environment, as well as the overall improvements in documentation, installation, and clarity greatly support the aforementioned claims. It plays nicely with several versions of Genologics LIMS, the current commercial leader, and therefore MetaLIMS does not 'lock' users into it like other LIMS systems do. The authors also include a complete database ER diagram, which was appreciated (possibly not necessary for the publication itself, but definitely should feature in documentation).

The following suggestions are speaking from a slightly biased perspective as one of the developers on MISO LIMS, in table 1. for MISO LIMS, sample metadata can be added at any time, but is limited to pre-defined fields and free-form "notes"; input sample types can be configured through advanced computer skills to include any type of sample (for OICR this starts at 'Tissue'); for software, Eclipse is a development environment, not a deployment software; finally, the correct website is http://tgac.github.io/miso-lims/ .

I support publication of this manuscript.

Level of Interest

Please indicate how interesting you found the manuscript: An article whose findings are important to those with closely related research interests

Quality of Written English

Please indicate the quality of language in the manuscript: Acceptable

Declaration of Competing Interests

Please complete a declaration of competing interests, considering the following questions:

- Have you in the past five years received reimbursements, fees, funding, or salary from an organisation that may in any way gain or lose financially from the publication of this manuscript, either now or in the future?
- Do you hold any stocks or shares in an organisation that may in any way gain or lose financially from the publication of this manuscript, either now or in the future?
- Do you hold or are you currently applying for any patents relating to the content of the manuscript?
- Have you received reimbursements, fees, funding, or salary from an organization that holds or has applied for patents relating to the content of the manuscript?
- Do you have any other financial competing interests?
- Do you have any non-financial competing interests in relation to this paper?

If you can answer no to all of the above, write 'I declare that I have no competing interests' below. If your reply is yes to any, please give details below.

6. I am involved with the development of MISO LIMS, a competing open source LIMS system mentioned in the paper. Although we develop MISO, I have no financial incentive to see other institutions adopt it.

I agree to the open peer review policy of the journal. I understand that my name will be included on my report to the authors and, if the manuscript is accepted for publication, my named report including any attachments I upload will be posted on the website along with the authors' responses. I agree for my report to be made available under an Open Access Creative Commons CC-BY license (http://creativecommons.org/licenses/by/4.0/). I understand that any comments which I do not wish to be included in my named report can be included as confidential comments to the editors, which will not be published.

I agree to the open peer review policy of the journal
